# Effects of bariatric surgery and dietary interventions for obesity on brain neurotransmitter systems and metabolism: A systematic review of positron emission tomography (PET) and single‐photon emission computed tomography (SPECT) studies

**DOI:** 10.1111/obr.13620

**Published:** 2023-09-12

**Authors:** Alhanouf S. Al‐Alsheikh, Shahd Alabdulkader, Alexander D. Miras, Anthony P. Goldstone

**Affiliations:** ^1^ Department of Metabolism, Digestion and Reproduction, Imperial College London Hammersmith Hospital London UK; ^2^ Department of Community Health Sciences, College of Applied Medical Sciences King Saud University Riyadh Saudi Arabia; ^3^ Department of Health Sciences, College of Health and Rehabilitation Sciences Princess Nourah Bint Abdulrahman University Riyadh Saudi Arabia; ^4^ School of Medicine, Faculty of Life and Health Sciences Ulster University Londonderry UK; ^5^ PsychoNeuroEndocrinology Research Group, Division of Psychiatry, Department of Brain Sciences, Imperial College London Hammersmith Hospital London UK

**Keywords:** dopamine, gastric bypass, opioid, sleeve gastrectomy

## Abstract

This systematic review collates studies of dietary or bariatric surgery interventions for obesity using positron emission tomography and single‐photon emission computed tomography. Of 604 publications identified, 22 met inclusion criteria. Twelve studies assessed bariatric surgery (seven gastric bypass, five gastric bypass/sleeve gastrectomy), and ten dietary interventions (six low‐calorie diet, three very low‐calorie diet, one prolonged fasting). Thirteen studies examined neurotransmitter systems (six used tracers for dopamine DRD2/3 receptors: two each for ^11^C‐raclopride, ^18^F‐fallypride, ^123^I‐IBZM; one for dopamine transporter, ^123^I‐FP‐CIT; one used tracer for serotonin 5‐HT_2A_ receptor, ^18^F‐altanserin; two used tracers for serotonin transporter, ^11^C‐DASB or ^123^I‐FP‐CIT; two used tracer for μ‐opioid receptor, ^11^C‐carfentanil; one used tracer for noradrenaline transporter, ^11^C‐MRB); seven studies assessed glucose uptake using ^18^F‐fluorodeoxyglucose; four studies assessed regional cerebral blood flow using ^15^O‐H_2_O (one study also used arterial spin labeling); and two studies measured fatty acid uptake using ^18^F‐FTHA and one using ^11^C‐palmitate. The review summarizes findings and correlations with clinical outcomes, eating behavior, and mechanistic mediators. The small number of studies using each tracer and intervention, lack of dietary intervention control groups in any surgical studies, heterogeneity in time since intervention and degree of weight loss, and small sample sizes hindered the drawing of robust conclusions across studies.

**Abbreviations**
VSG: vertical sleeve gastrectomy
RYGB: Roux‐en‐Y gastric bypass
PET: positron emission tomography
SPECT: single‐photon emission computed tomography
fMRI: functional magnetic resonance imaging
BGU: brain glucose uptake
rCBF: regional cerebral blood flow
BMI: body mass index
NIH: National Institutes for Health
VLCD: very low calorie diet
LCD: low‐calorie diet
T2DM: type 2 diabetes mellitus
BP: binding potential
^123^I‐IBZM: ^123^I‐iodobenzamide
DRD2/3: dopamine D2/3 receptors
^123^I‐FP‐CIT: ^123^I‐N‐ω‐fluoropropyl‐2β‐carbomethoxy‐3β‐(4‐iodophenyl) nortropane
^11^C‐PHNO: ^11^C‐4‐propyl‐9‐hydroxynaphthoxazine
DAT: dopamine transporter
5‐HT: serotonin
5‐HT_2C_R: serotonin 2C receptor
5‐HT_2A_R: serotonin 2A receptor
SERT: serotonin transporter
^11^C‐DASB: ^11^C‐3‐amino‐4‐(2‐dimethylaminomethyl‐phenylsulfanyl)‐benzonitrile
MOR: μ‐opioid receptors
ROIs: regions of interest
NAT: noradrenaline transporter
^11^C‐MRB: ^11^C‐methylreboxetine
^18^F‐FTHA: ^18^F‐fluoro‐6‐thia‐heptadecanoic acid
^15^O‐H_2_O: ^15^O‐water
ASL: arterial spin labeling
GLP‐1: glucagon‐like peptide‐1
PYY: peptide YY
FPG: fasting plasma glucose
FFA: free fatty acid
aROIs: anatomical regions of interest

## 1 BACKGROUND

### 1.1 Introduction

In many parts of the world, obesity has reached pandemic proportions; the number of deaths because of obesity‐related health issues is rising at an unprecedented pace, and controlling obesity remains a daunting challenge. The obesity epidemic has tripled since 1975; in 2016, 39% of adults had overweight, and 13% had obesity globally.^1^ The last report from the National Health Service in 2020 estimated that obesity might affect one in every four adults in the United Kingdom (25% of the population).^2^

*Obesity surgery* is the most effective long‐term treatment for obesity.^3^, ^4^ As the number of obesity surgery operations has increased in the last decade, elucidating the mechanisms of action is crucial and a key research goal that may help optimize surgical outcomes by improving patient selection.^5^ Moreover, understanding the mechanism of action by which each procedure reduces energy intake may eventually facilitate novel non‐surgical approaches, including medications.^3^, ^5^ Vertical sleeve gastrectomy (VSG) and Roux‐en‐Y gastric bypass (RYGB) are currently the most commonly performed obesity surgeries worldwide.^4^, ^6^ Both procedures result in sustained weight reduction with no significant difference in terms of weight loss (20–25%) between the two groups after 5 years post‐surgery.^7^, ^8^ Although both operations decrease gastric volume, the changes in appetitive gut hormones reduce energy intake by affecting the brain, which produces sustained weight loss.^3^ Moreover, changes in taste, food preference, food hedonics, and food cue reactivity have been seen in some studies after RYGB and VSG surgery.^5^, ^9^ However, this depends on the particular outcome measures used. After bariatric surgery, reductions in food cue reactivity in brain reward systems using functional magnetic resonance imaging (fMRI) paradigms, motivation to work, and liking and wanting of high‐energy (HE) over low‐energy (LE) foods have been found, though preferential reductions in actual intake of HE over LE foods in the laboratory setting have not been reported.^5^, ^10^, ^11^, ^12^, ^13^, ^14^, ^15^, ^16^, ^17^, ^18^, ^19^, ^20^ In patients with obesity, hyperactivity of the brain in areas associated with reward and hypoactivity in areas associated with cognitive control have been reported.^21^, ^22^, ^23^, ^24^

*Non‐surgical interventions* usually consist of dietary changes and behavioral therapy, with the primary goal of reducing energy intake, increasing physical activity, and various pharmacotherapies.^4^ Although non‐surgical interventions may achieve weight loss, most of the non‐pharmacotherapy methods lead to weight regain over the long‐term because of compensatory adaptations in body weight regulation, which promote rapid weight regain efficiently.^25^

*Functional neuroimaging techniques*, such as positron emission tomography (PET), single‐photon emission computed tomography (SPECT), fMRI, magnetoencephalography, and electroencephalography, are recently developed tools to investigate the brain centers involved in the control of appetite signals, eating behavior, and the pathophysiology of obesity.^26^ These techniques offer insight into the brain by providing objective and sensitive information, accelerating scientific progress, and facilitating hypothesis testing.^27^ In brief, PET is an imaging technique that provides semi‐quantitative and quantitative measurements of biochemical processes by measuring the density of various neuroreceptor subtypes. These neuroreceptors include dopamine, opioids, noradrenaline, and serotonin.^28^ PET also measures the transporter availability of certain neurotransmitters and physiological process including measurement of the brain glucose uptake (BGU), fatty acid uptake, and regional cerebral blood flow (rCBF) which reflect local neuronal activity.^26^ These measurements rely partly on the use of a pharmacological or physiological compound labeled with a positron‐emitting radioisotope, such as ^18^F, ^11^C, and ^15^O.

Like PET, SPECT is another imaging method providing information about biochemical and physiological processes. SPECT radiotracers are elements or pharmacological compounds that include radioactive isotopes such as iodine‐123 (^123^I).^26^ Only PET and SPECT can provide information on a molecular level because specific molecules can be labeled to allow their detection.^29^

This systematic review will discuss how these neural systems are dysregulated in human obesity and the effects of dietary and surgical weight loss interventions. This will help understand the mechanisms that lead to overeating and the development of obesity, and the mechanisms behind weight loss, by comparing the differences post‐intervention with pre‐intervention, or participants with versus without obesity, in brain area related to reward processing, homeostatic control of eating behavior, inhibitory control, executive function, and cognition. Moreover, it evaluates the association of changes in brain tracer uptake with clinical outcomes, behavioral changes, and appetitive gut hormones.

To our knowledge, there is no systematic review that has previously investigated the effect of surgical and other non‐pharmacological interventions on the brain, other than one conducted in 2013 that examined the impact of obesity surgery on the brain which included only three PET studies (19 PET/SPECT studies have been conducted after 2013).^30^ Therefore, this systematic review will identify all the available evidence to evaluate and summarize the finding and help identify any literature gaps.

### 1.2 Aims and objectives

- Identify PET or SPECT studies in patients with overweight/obesity examining effects of bariatric surgery or dietary interventions in longitudinal or cross‐sectional design.
- Summarize and critically review the findings from the studies identified.
- Examine the following issues:
  - how heterogeneity in study design, methodology, protocol, and analysis might explain discrepancies between studies.
  - associations of brain PET/SPECT findings with clinical outcomes, eating behavior measures, and potential mechanistic mediators, for example, gut hormones. This review includes predictive studies that focus on assessing the effects of an intervention on clinical outcomes, eating behavior measures, and potential mechanistic mediators. Cross‐sectional studies that looked only at correlations among PET/SPECT and clinical features, eating behavior measures and mechanistic mediators, in participants *before* any intervention, and studies that only looked at pharmacological interventions are out of the scope of this review.

A systematic review was completed of studies investigating the impact of bariatric surgery and dietary intervention on brain function using PET/SPECT scans. A comprehensive search of the literature was undertaken to obtain information on both longitudinal and cross‐sectional human studies.

## 2 METHODS

### 2.1 Inclusion and exclusion criteria

The studies selected for the review included the following criteria.

#### 2.1.1 Inclusion criteria

- Longitudinal and cross‐sectional human studies.
- Studies published in English.
- Articles published between January 1980 and April 2021.
- Studies conducted on adolescents or adults aged 16 years and older, of either sex.
- Participants in the intervention group should be diagnosed with overweight (body mass index, BMI > 25 kg/m^2^) or obesity with BMI > 30 kg/m^2^.
- Assessments of obesity surgery (RYGB, VSG, one anastomosis gastric bypass, gastric banding, vertical band gastroplasty, biliary‐pancreatic diversion, and gastric balloon) and dietary interventions.
- Studies using brain PET/SPECT scanning, including tracers assessing neurotransmitter systems, rCBF, glucose uptake, or uptake of other metabolites.

#### 2.1.2 Exclusion criteria

- Studies performed on children <16 years old.
- Studies conducted on animals.
- Reviews and meetings abstracts.
- PET/SPECT studies that just assessed the impact of interventions on peripheral tracer binding (such as the heart, gastrointestinal tract, or adipose tissue).
- PET/SPECT studies that only included a pharmacological intervention.

### 2.2 Database search

An electronic database search was performed to find the articles to form the evidence base for this review. A comprehensive search was performed across multiple databases and journals using PubMed, Web of Science, PsycINFO, MEDLINE, and EMBASE databases within OVID. Reference lists were also examined from individual papers and relevant review articles.

#### 2.2.1 Keywords/terms used

The detailed keywords and terms used are provided in Data S1 Methods.

### 2.3 Data extraction

A complete description of all data extraction is available in Data S1 Methods.

### 2.4 Methodological quality assessment

The reviewer assessed the methodological quality of the articles by using the National Institutes of Health (NIH) Quality Assessment Tool for the following: (i) observational cohort and cross‐sectional studies, (ii) before–after (pre‐post) studies with no control group, (iii) controlled intervention studies (https://www.nhlbi.nih.gov/health-topics/study-quality-assessment-tools), including appraisal criteria specific to the study design. For instance, studies were rated based upon the following criteria: exposure‐related considerations (timeline relative to outcome measurement, frequency of measure, and categorization of exposure levels); methodological validity of exposure and outcome measurements; participation and post‐baseline follow‐up rates; adjustment for confounding variables; outcome assessor blinding; and explicitness of aims, sample, and study setting. The summary score of each study was calculated based on applicable questions for that particular study, expressed as a percentage ranging from 0% to 100%. These were categorized into three categories of quality assessment: poor (0–33.3%), fair (33.4–66.6%), good (66.7–100%), which were equated to high, low, and very low risk of bias.^31^

## 3 RESULTS

### 3.1 Search results and selection of studies

**FIGURE 1:**
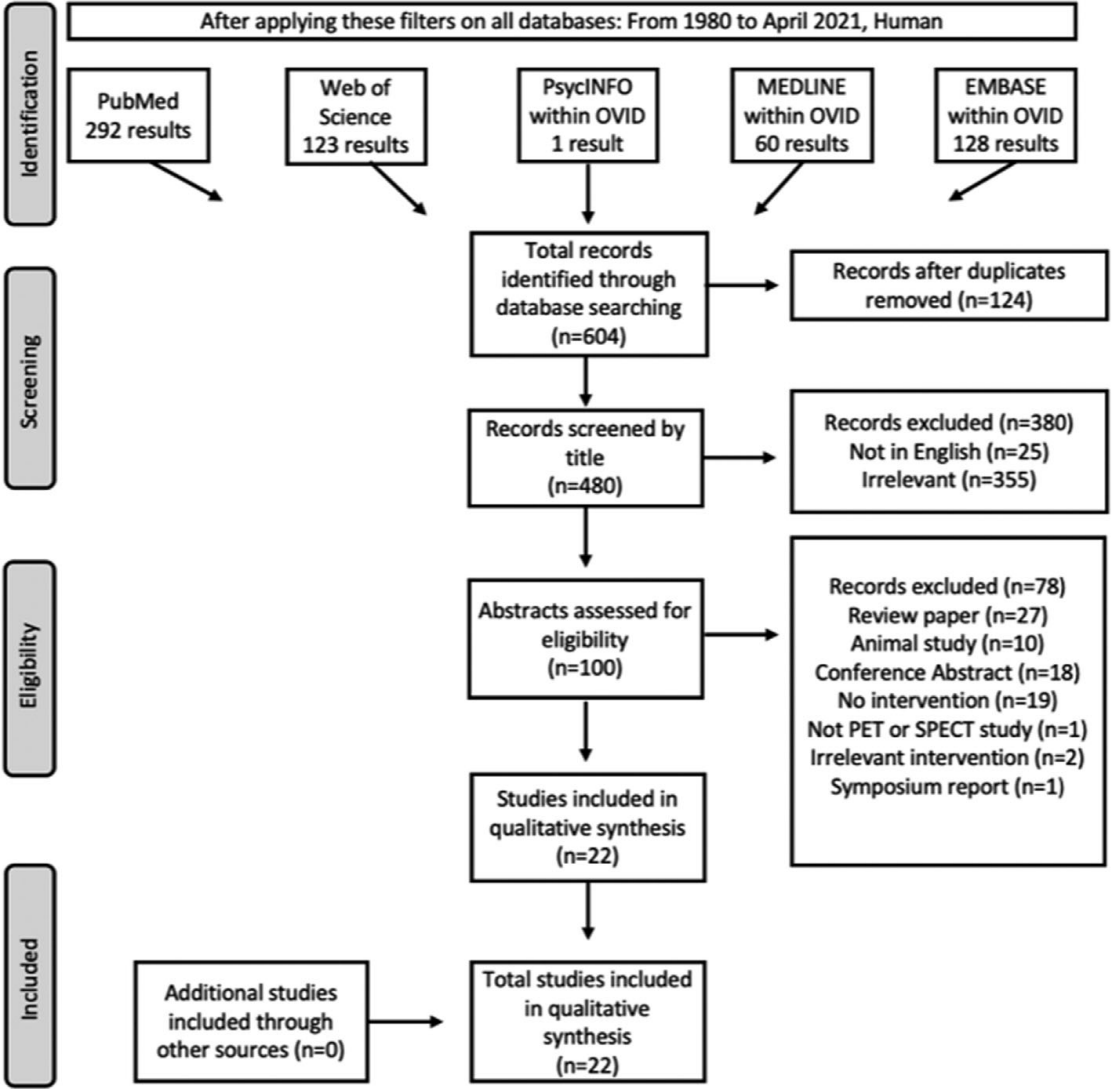
PRISMA flow diagram for included studies.

Using the keywords, 604 articles were identified and 480 of these were screened after duplicates were removed. From these articles, 458 were excluded with only 22 studies meeting the inclusion criteria (Figure 1).

Nineteen of these studies used PET scans,^32^, ^33^, ^34^, ^35^, ^36^, ^37^, ^38^, ^39^, ^40^, ^41^, ^42^, ^43^, ^44^, ^45^, ^46^, ^47^, ^48^, ^49^, ^50^ whereas three studies used SPECT scans.^51^, ^52^, ^53^

### 3.2 Study summary

#### 3.2.1 PET/SPECT tracers

**FIGURE 2:**
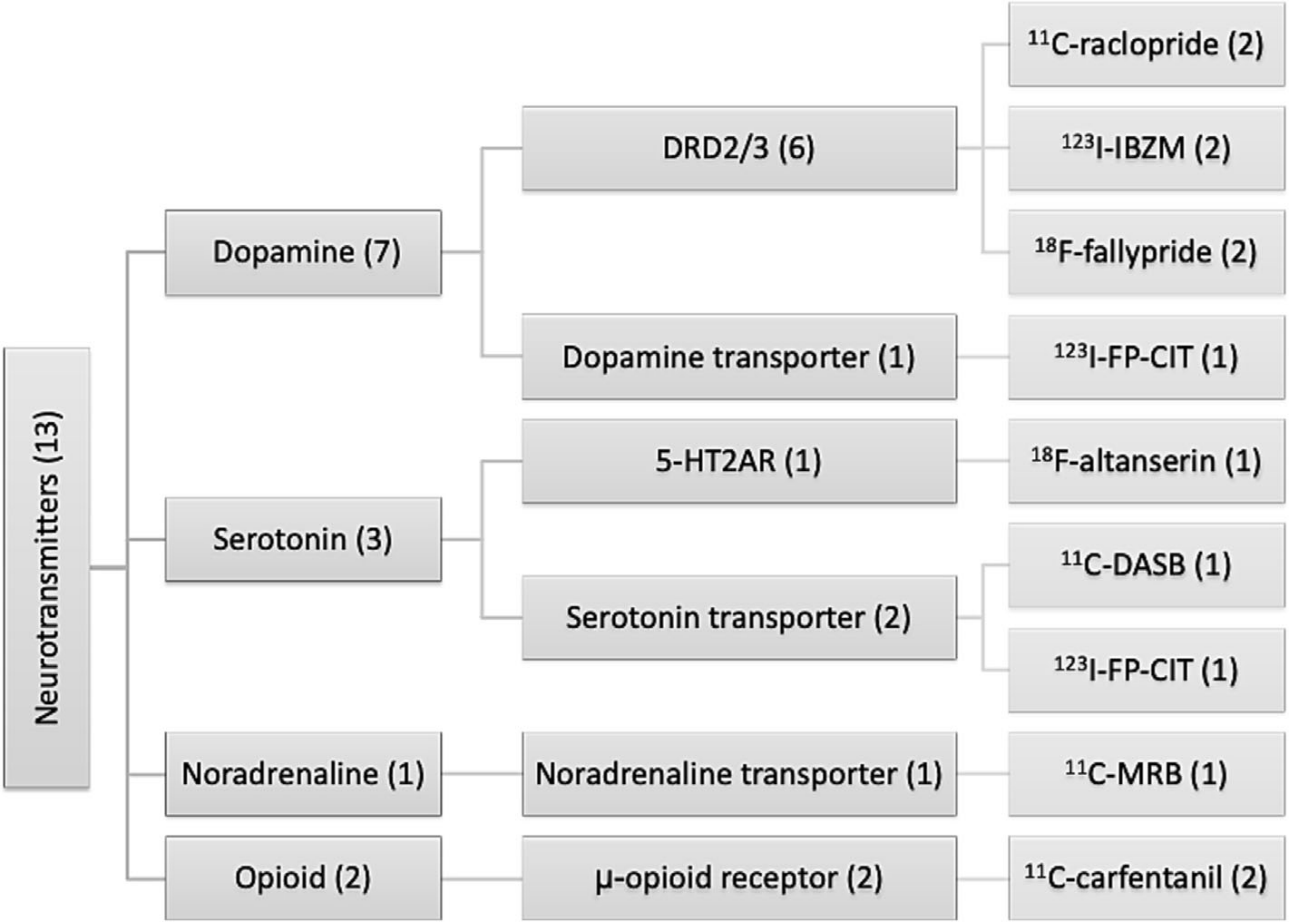
Summary of radioactive tracers used to investigate neurotransmitter systems. Number in brackets indicates number of studies.

**FIGURE 3:**
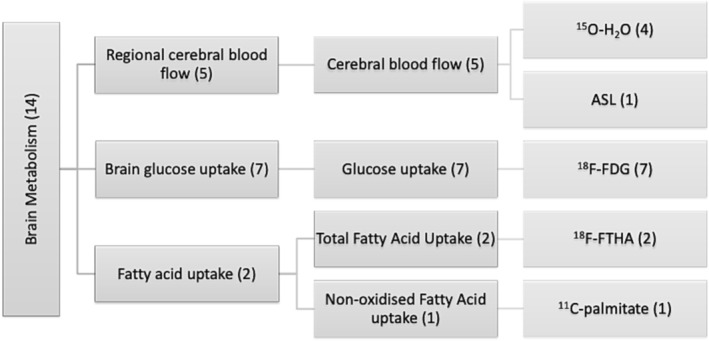
Summary of radioactive tracers used to investigate brain metabolism. Number in brackets indicates number of studies.

A complete description of all PET/SPECT tracers is available in S1 Results. Radioactive tracers used to investigate neurotransmitter systems are illustrated in Figure 2. Radioactive tracers used to investigate brain metabolism are illustrated in Figure 3.

#### 3.2.2 Country

The country where the studies were conducted are provided in Table 1 and summarised in Data S1 Results: 3.2.2. Country.

#### 3.2.3 Study design

Study summaries are presented in Table 1.

**TABLE 1:** Study summaries.

Author, year	Journal	Country	Tracer	Target	Design	Bariatric surgery	Non‐surgical dietary intervention	Control group	Group (s)	Task	Paradigm	Nutritional state interaction	Other state intervention	Association PET/SPECT with clinical outcome	Appetite ratings	Other eating behavior measures	Association PET/SPECT with appetite/behavior	Assessment nausea or dumping symptoms	Mechanistic blood measures	Association PET/SPECT with mechanistic measures	Exclusion criteria: use of psychotropic medication
**DOPAMINE**																					
^**11**^ **C‐raclopride**																					
Steele, 2010	Obes Surg	USA	^11^C‐raclopride	DRD2/3	long.	Yes (mix)	o	Yes (CS)	RYGB, NWC	o	n/a	o	o	o	o	o	o	o	o	o	Yes
Karlsson, 2016^a^	Mol Psychiatry	Finland	^11^C‐raclopride	DRD2/3	long.	Yes (mix)	o	Yes (CS)	RYGB/VSG, NOC	o	n/a	o	o	Yes	o	Yes	Yes	o	Yes	Yes	Yes
^**18**^ **F‐fallypride**																					
Dunn, 2010	Brain Res	USA	^18^F‐fallypride	DRD2/3	long.	Yes (mix)	o	o	RYGB/VSG	o	n/a	o	o	o	o	Yes	o	o	Yes	o	Yes
Dunn, 2017	Obesity	USA	^18^F‐fallypride	DRD2/3	long.	o	Yes	o	OB‐VLCD	o	n/a	o	o	o	o	o	o	o	Yes	Yes	Yes
^**123**^ **I‐IBZM**																					
de Weijer, 2014^b^	Diabetologia	Netherlands	^123^I‐IBZM	DRD2/3	long.	Yes	o	o	RYGB	o	n/a	o	o	Yes	o	o	o	o	Yes	Yes	Yes
van der Zwaal, 2016^b^	Eur Neuropsychopharmacol	Netherlands	^123^I‐IBZM	DRD2/3	long.	Yes	o	Yes (CS)	RYGB, NOC	o	n/a	o	o	Yes	o	Yes	Yes	o	Yes	Yes	Yes
^**123**^ **I‐FP‐CIT**																					
Versteeg, 2017^c^	FASEB J	Netherlands	^123^I‐FP‐CIT	DAT	long.	o	Yes	o	OB‐LCD‐BR, OB‐LCD‐D^d^	o	n/a	o	o	o	Yes	o	o	o	Yes	o	Yes
**SEROTONIN**																					
^**123**^ **I‐FP‐CIT**																					
Versteeg, 2017^c^	FASEB J	Netherlands	^123^I‐FP‐CIT	SERT	long.	o	Yes	o	OB‐LCD‐BR, OB‐LCD‐D^d^	o	n/a	o	o	o	Yes	o	o	o	Yes	o	Yes
^**18**^ **F‐altanserin and** ^**11**^ **C‐DASB**																					
Haahr, 2015	J Neurosci	Denmark	^11^C‐DASB, ^18^F‐altanserin	SERT, 5‐HT_2A_R	long.	Yes	o	Yes (CS)	RYGB, NWC	o	n/a	o	o	Yes	Yes	o	Yes	o	Yes	Yes	Yes
**NORADRENALINE**																					
^**11**^ **C‐MRB**																					
Vettermann, 2018	Eur J Nucl Med Mol Imaging	Germany	^11^C‐MRB	NAT	long.	o	Yes	Yes (CS)	OB‐LCD, NOC‐NT	o	n/a	o	o	Yes	o	Yes	o	o	o	o	Yes
**OPIOID**																					
^**11**^ **C‐carfentanil**																					
Karlsson, 2016^a^	Mol Psychiatry	Finland	^11^C‐carfentanil	MOR	long.	Yes (mix)	o	Yes (CS)	RYGB/VSG, NOC	o	n/a	o	o	Yes	o	Yes	Yes	o	Yes	Yes	Yes
Burghardt, 2015	J Clin Endocrinol Metab	USA	^11^C‐carfentanil	MOR	long.	o	Yes	Yes (CS)	OB‐VLCD, NWC	o	n/a	Yes	o	Yes	Yes	o	Yes	o	o	o	Yes
**GLUCOSE METABOLISM**																					
^**18**^ **F‐FDG**																					
Hunt, 2016	Diab Care	UK	^18^F‐FDG	GU	CS	Yes	o	Yes (CS)	RYGB, OB, NWC	o	n/a	Yes	± SST/insulin infusion	o	Yes	Yes	Yes	Yes	Yes	Yes	Yes
Rebelos, 2019	Diabetes Obes Metab	Finland	^18^F‐FDG	GU	long.	Yes (mix)	o	Yes (CS)	RYGB/VSG, NOC	o	n/a	o	± HEC	Yes^h^	o	o	o	o	Yes	Yes^h^	Yes
Marques, 2014	J Clin Endocrinol Metab	Brazil	^18^F‐FDG	GU	long.	Yes	o	Yes (CS)	RYGB, NWC	o	n/a	o	o	o	o	o	o	o	Yes	o	Yes
Tuulari, 2013	Diabetes	Finland	^18^F‐FDG	GU	long.	Yes (mix)	o	Yes (CS)	RYGB/VSG, NOC	o	n/a	o	o	o	o	o	o	o	Yes	o	Yes
Guzzardi, 2018	Eur Eat Disord Rev	Italy	^18^F‐FDG	GU	long.	o	Yes	o	OW‐LCD (low vs. high YFAS)^e^	Yes	Food cue reactivity, Taste, Food odor	o	o	Yes	Yes	Yes	Yes	o	Yes	Yes	Yes
Redies, 1989^a^	Am J Physiol	Canada	^18^F‐FDG	GU	long.	o	Yes	o	OB‐fast^f^	o	n/a	o	o	o	o	o	o	o	Yes	o	o
Almby, 2021	Diabetes	Sweden	^18^F‐FDG	GU	long.	Yes	o	o	RYGB	o	n/a	o	HEC vs. HOC	o	o	o	o	o	Yes	o	Yes
**CEREBRAL BLOOD FLOW**																					
^**15**^ **O‐H**_**2**_ **O**																					
Redies, 1989^a^	Am J Physiol	Canada	^15^O‐H_2_O	CBF	long.	o	Yes	o	OB‐fast	o	n/a	o	o	o	o	o	o	o	Yes	o	o
Delparigi, 2004^b^	Int J Obesity	USA	^15^O‐H_2_O	CBF	CS	o	Yes	Yes (CS)	post‐OB‐LCD^j^, OB, NWC	o	Taste	Yes	o	o	Yes	o	Yes	o	Yes	Yes	Yes
Delparigi, 2007^b^	Int J Obesity	USA	^15^O‐H_2_O	CBF	CS	o	Yes	Yes (CS)	post‐OB‐LCD^j^, OB	o	Taste	Yes	o	o	Yes	Yes	Yes	o	Yes	Yes	Yes
Le, 2007^b^	Am J Clin Nutr	USA	^15^O‐H_2_O	CBF	CS	o	Yes	Yes (CS)	post‐OB‐LCD^j^, OB, NWC	o	o	Yes	o	o	Yes	o	o	o	Yes	o	Yes
**ASL**																					
Almby, 2021	Diabetes	Sweden	ASL	CBF	long.	Yes	o	o	RYGB	o	n/a	o	HEC vs. HOC	o	o	o	o	o	Yes	o	Yes
**FATTY ACID METABOLISM**																					
^**18**^ **F‐FTHA and** ^**11**^ **C‐palmitate**																					
Karmi, 2010	Diabetes	Finland	^18^F‐FTHA, ^11^C‐palmitate	total FAU non‐oxidized FAU	long.	o	Yes	Yes (CS)	MS‐VLCD, NOC	o	n/a	o	o	Yes	o	o	o	o	Yes	o	Yes
Rebelos, 2020	Diabetes Obes Metab	Finland	^18^F‐FTHA	total FAU	long.	Yes (mix)	o	Yes (CS)	RYGB/VSG, NOC	o	n/a	o	o	Yes	o	o	o	o	Yes	Yes	Yes

Abbreviations: ^11^C‐DASB, ^**11**^C‐3‐amino‐4‐(2‐dimethylaminomethyl‐phenylsulfanyl)‐benzonitrile; ^11^C‐MRB, ^11^C‐methylreboxetine; ^123^I‐FP‐CIT, ^123^I‐N‐ω‐fluoropropyl‐2β‐carbomethoxy‐3β‐(4‐iodophenyl)nortropane; ^123^I‐IBZM, ^123^I‐iodobenzamide; ^15^O‐H_2_O, ^15^O‐water; ^18^F‐FDG, ^18^F‐fluorodeoxyglucose; ^18^F‐FTHA, ^18^F‐fluoro‐6‐thia‐heptadecanoic acid; 5‐HT_2A_R, serotonin 2A receptor; ASL, arterial spin labeling; BMI, body mass index; BR, breakfast; CBF, cerebral blood flow; CHO, carbohydrate; CS, cross‐sectional; D, dinner; DAT, dopamine transporter; DRD2/3, dopamine receptor D2/3; FAU, fatty acid uptake; GU, glucose uptake; HEC, hyperinsulinemic euglycemic clamp; HOC, hyperinsulinemic hypoglycemic clamp; LCD, low‐calorie diet; long., longitudinal; mix, mixed group; MOR, μ‐opioid receptor; MS, metabolic syndrome; n/a, not applicable; NAT, noradrenaline transporter; NOC, non‐obese control; NT, no treatment; NWC, normal weight control (lean); o, no; OB, obesity; OW, overweight; PET, positron emission tomography; REE, resting energy expenditure; RYGB, Roux‐en‐Y gastric bypass; SERT, serotonin transporter; SPECT, single‐photon emission computerized tomography; SST, somatostatin; UK, United Kingdom; USA, United States of America; VLCD, very low‐calorie diet; VSG, vertical sleeve gastrectomy; YFAS, Yale Food Addiction Scale.

^a^ Same datasets.

^b^ Overlapping datasets.

^c^ Same dataset and tracer (SERT binding at 2 h, DAT binding at 3 h).

^d^ 50% of total 24‐h energy requirements (calculated from 1.33 × REE using indirect calorimetry) with 35% at lunch, and either 50% at breakfast, 15% at dinner (LCD‐BR) or 15% at breakfast, 50% at dinner (LCD‐D).

^e^ 1600 kcal/day (30% fat, 50% CHO, 20% protein).

^f^ Fasted for 3 weeks.

^g^ With diet and exercise BMI fallen from >35 to ≤25 kg/m^2^ and weight stable ≥ 3 months.

^h^ But no localization reported.

Of the included studies, 18 (81.8%) were of a longitudinal design^32^, ^33^, ^34^, ^35^, ^36^, ^37^, ^39^, ^40^, ^41^, ^42^, ^43^, ^46^, ^47^, ^49^, ^50^, ^51^, ^52^, ^53^ with 11 of these (61.1%) including a surgical intervention and seven (38.9%) a dietary intervention. No studies included a control dietary intervention in the same publication as the surgical intervention. Out of the four (18.2%) cross‐sectional studies, one included a surgical intervention^38^ and three a dietary intervention.^44^, ^45^, ^48^

Among the different types of interventions, 12 studies (54.5%) assessed the effect of surgery: seven included RYGB surgery^32^, ^37^, ^38^, ^40^, ^50^, ^51^, ^52^ and five included a mixed RYGB/VSG surgery group.^33^, ^34^, ^39^, ^41^, ^49^ There were no studies that assessed only VSG surgery and no studies included one anastomosis gastric bypass, gastric banding, biliopancreatic diversion or gastric balloon.

Among the 10 studies (45.5%) assessing dietary interventions, three included very low‐calorie diet (VLCD),^35^, ^36^, ^46^ six low‐calorie diet (LCD),^42^, ^44^, ^45^, ^47^, ^48^, ^53^ and one study assessed total fasting for 3 weeks.^43^

### 3.3 Demographic data

Demographic data for individual studies are given in Table 2.

**TABLE 2:** Demographic data.

Author, year	*N*	Group (s)	Female	Age at baseline (y)	T2DM	White Caucasian	Control intervention	Time scan pre‐intervention (months)	Time between scans (months)	Time scan post‐intervention (months)	Baseline BMI	Current/post‐BMI (kg/m2)	Weight loss	Change in glycaemia
			*n* (%)	Mean ± SD or median [IQR] (range)	*n* (%)	*n* (%)				Mean ± SD or median [IQR] (range)	Mean ± SD or median [IQR] (range)	Mean ± SD or median [IQR] (range) kg/m^2^	Mean ± SD (range) % or kg	Mean ± SD
**DOPAMINE**														
^**11**^ **C‐raclopride**														
Steele, 2010	5	RYGB	5 (100%)	32.2 ± 7.3 (20–38)	0 (0%)	2 (40.0%)	n/a	?	?	(0.9–1.4)	45.2 ± 5.9 (40–53)	38.0 ± 6.9	12.9 ± 6.5% (6.5–23.0)^t^	?
	5	NWC	5 (100%)	21.8	0 (0%)	?	None	n/a	n/a	n/a	21.3	n/a	n/a	n/a
Karlsson, 2016^a^	16 (? RYGB, ? VSG)	RYGB/VSG	16 (100%)	42.8 ± 10.2	6 (37.5%)	?	n/a	pre‐VLCD	?	6	40.3 ± 3.9 (36.1–49.3)	31.0 ± 3.7	~23.3%^u^	HbA1c (%): ↓ pre‐RYGB: 5.9 ± 0.8, post‐RYGB: 5.4 ± 0.5
	14	NOC	14 (100%)	44.9 ± 12.9	0 (0%)	?	None	n/a	n/a	n/a	22.7 ± 2.9	n/a	n/a	
^**18**^ **F‐fallypride**														
Dunn, 2010	5 (4 RYGB, 1 VSG)	RYGB/VSG	5 (100%)	45.8 ± 4.3 (41–50)	0 (0%)	4 (80%)	n/a	?	median 2.1 (1.8–5.3)	median 1.6 (1.4–2.5)	43.2 ± 6.3 (38–54)	38 ± 7	11.6 ± 2.0% (8.5–13.4)^u^	?
Dunn, 2017	15	OB‐VLCD^m^	15 (100%)	39 ± 8	1 (6.7%)	8 (53.3%)	n/a	0	(0.26–0.32)	(0.26–0.32)	39 ± 6	38 ± 6	~2.9%^u^	FPG (mmol/L): ↓
^**123**^ **I‐IBZM**														
de Weijer, 2014^b^	19	RYGB	19 (100%)	40.4 ± 8 (26–49)	?	19 (100%)	n/a	?	?	1.4	45.7 ± 6.3 (38.7–1.3)	40.9 ± 6.3 (34.1–57.6)	14 ± 4.6 kg (8–24)	?
van der Zwaal, 2016^b^	11 (14 overall)^d^	RYGB	11 (100%), overall 14 (100%)	44.3 ± 6	?	11 (100%)	n/a	?	?	37.2 (25.2–43.2)^g^	45.2 ± 6.7 (38.7–61.3)^g^	31.2 ± 5.7 (24.1–43.7)^g^	~30.9%^t^ ^,^ ^g^	FPG (mmol/L): ↓ pre‐RYGB: 5.6 ± 0.8, post‐RYGB: 4.6 ± 0.2 g
	11	NOC	11 (100%)	40.5 ± 4	?	11 (100%)	None	n/a	n/a	n/a	21.9 ± 2.0	n/a	n/a	
^**123**^ **I‐FP‐CIT**														
Versteeg, 2017^c^	9 (12 overall)^e^	OB‐LCD‐BR^n^	0 (0%), 0 (0%)	60.7 ± 7.7^j^	0 (0%) but 100% IFG or IR	?	n/a	0	0.9	0.9	34.2 ± 4.2^j^	?	6.5 ± 1.5%^u^	?
	11	OB‐LCD‐D^n^	0 (0%)	59.0 ± 8.5	0 (0%) but 100% IFG or IR	?	n/a	0	0.9	0.9	34.3 ± 3.7	?	6.2 ± 1.9%^u^	?
**SEROTONIN**														
^**123**^ **I‐FP‐CIT**														
Versteeg, 2017^c^	9 (12 overall)^e^	OB‐LCD‐BR^n^	0 (0%), overall 0 (0%)	60.7 ± 7.7^e^	0 (0%) but 100% IFG or IR	?	n/a	0	0.9	0.9	34.2 ± 4.2^e^	?	6.5 ± 1.5%^u^	?
	11	OB‐LCD‐D^n^	0 (0%)	59.0 ± 8.5	0 (0%) but 100% IFG or IR	?	n/a	0	0.9	0.9	34.3 ± 3.7	?	6.2 ± 1.9%^u^	?
^**18**^ **F‐altanserin and** ^**11**^ **C‐DASB**														
Haahr, 2015	pre‐RYGB: 20–21, post‐RYGB: 12–13	RYGB	pre‐RYGB: 16–17 (80.0–80.9%), post‐RYGB: 10–11 (83.3–84.6%)	41.3 ± 8.4^s^	?	?	n/a	3.1 [1.8–6.0]	?	8.2 [7.5–8.5]	40.1 ± 4.1	28.9 ± 4.1	25.80%	?
	10	NWC	7 (70.0%)	45.6 ± 9.7	0 (0%)	?	None	n/a	n/a	n/a	24.6 ± 1.5	n/a	n/a	n/a
**NORADRENALINE**														
^**11**^ **C‐MRB**														
Vettermann, 2018	10	OB‐LCD	4 (40.0%)	34.4 ± 9.0	0 (0%)	10 (100%)	n/a	?	6.7 ± 1.5	6	42.4 ± 3.7	41.0 ± 3.8	3.7%^u^	?
	9 (10 overall)^f^	NOC‐NT	? (?%), overall 4 (40.0%)^f^	33.3 ± 10.0^f^	0 (0%)	10 (100%)^l^	None	n/a	6.7 ± 1.6	6	23.9 ± 2.5^f^	23.8 ± 2.5^f^	~0.5%^u^	n/a
**OPIOID**														
^**11**^ **C‐carfentanil**														
Karlsson, 2016^a^	16 (? RYGB, ? VSG)	RYGB/VSG	16 (100%)	42.8 ± 10.1	6 (37.5%)	?	n/a	pre‐VLCD	?	6	40.3 ± 3.9 (36.1–49.3)	31.0 ± 3.7	~23.3%^u^	HbA1c (%):↓ pre‐RYGB: 5.9 ± 0.8, post‐RYGB: 5.4 ± 0.5
	14	NOC	14 (100%)	44.9 ± 12.9	0 (0%)	?	None	n/a	n/a	n/a	22.7 ± 2.9	n/a	n/a	
Burghardt, 2015	6 (7 overall)^g^	OB‐VLCD^o^	0 (0%), overall 0 (0%)	51.4 ± 11.2^g^	?	?	n/a	?	?	3.6 ± 0.7 (2.9–4.5)	38.0 ± 3.4^g^	31.8 ± 1.8	~16.2%^u^	?
	7	NWC	0 (0%)	52.4 ± 9.0	?	?	None	n/a	n/a	n/a	24.0 ± 1.7	n/a	n/a	
**GLUCOSE METABOLISM**														
^**18**^ **F‐FDG**														
Hunt, 2016	9	RYGB	8 (88.9%)	45.1 ± 10.7	? (1 on metformin)	5 (55.6%)	n/a	n/a	n/a	18 ± 12.6	?	34.0 ± 3.3	30.9 ± 8.5%	n/a
	21	OB	19 (90.5%)	31.1 ± 10.5	? (1 on metformin)	14 (66.7%)	n/a	n/a	n/a	n/a	n/a	34.1 ± 2.6	n/a	
	12	NWC	9 (75%)	32.3 ± 9.3	?	11 (91.7%)	n/a	n/a	n/a	n/a	n/a	22.3 ± 1.4	n/a	
Rebelos, 2019	16–20^h^ (11 RYGB, 9 VSG)	RYGB/VSG	16 (100%), overall 19 (95.0%)^h^	46 ± 9^h^	6 (31.6%)^h^	?	n/a	> 1	~7	6	43.1 [2.5]^h^	32.2 [3.1]	~26.1%^u^, ~25.3%^t^	T2DM: ↓ 30.0% to 6.3%
	12	NOC	8 (66.7%)	43 ± 11	0 (0%)	?	None	n/a	n/a	n/a	23.2 [3.0]	n/a	n/a	IGT: ↓ 50.0% to 12.5%
														HbA1c (%): ↓ pre: 5.8 ± 0.5, post: 5.5 ± 0.3
Marques, 2014	17	RYGB	17 (100%)	40.5 ± 10.1	0 (0%)	?	n/a	?	?	6	50.1 ± 4.7	37.2 ± 4.1	~25.7%^t^	FPG (mmol/L): ↓ pre: 5.4 ± 0.7, post: 4.7 ± 0.5
	16	NWC	16 (100%)	41.4 ± 8.7	0 (0%)	?	None	n/a	n/a	n/a	22.3 ± 2.1	n/a	n/a	
Tuulari, 2013	17–22^i^ (? RYGB, ? VSG)	RYGB/VSG	17 (100%), 20 (90.9%)	45.4 ± 9.3	4 (23.5%)	?	n/a	>1	>7	6	43.1 ± 3.0	33.2 ± 3.8	~23.3%^u^	HbA1c (%): ↓ pre: 5.8 ± 0.5 post: 5.5 ± 0.3
	7	NOC	5 (71.4%)	47.9 ± 5.6	0 (0%)	?	None	n/a	n/a	n/a	23.8 ± 2.1	n/a	n/a	FPG (mmol/L): ↓ pre: 6.2 ± 0.9, post: 5.3 ± 0.6
														T2DM: ↓ 23.5% to 17.6%
														IGT: ↓ 23.5% to 17.6%
Guzzardi, 2018	11–14^j^	OW‐LCD (low‐YFAS)^p^	11 (100%), overall 14 (100%)	33.8 ± 10.8	0 (0%)	?	n/a	0	3	3	32.9 ± 3.7	32.0 ± 4.0	4.6 ± 1.1%	HbA1c (%): pre: 5.4 ± 0.3, post: 5.4 ± 0.3
	12–22^j^	OW‐LCD (high‐YFAS)^p^	12 (100%), overall 22 (100%)	37.5 ± 8.9	0 (0%)	?	n/a	0	3	3	32.7 ± 3.3	31.8 ± 3.5	4.1 ± 1.2%	HbA1c (%): pre: 5.4 ± 3.3, post: 5.3 ± 0.4
Redies, 1989^a^	4	OB‐fast	0 (0%)	37.8 ± 6.2	0 (0%)	?	n/a	0	0.6–0.8	0.6–0.8	36.2 ± 4.1	?	11.8 ± 1.9%	FPG (mmol/L): ↓ pre: 5.4 ± 1.1, post: 4.1 ± 0.3
Almby, 2021	11	RYGB	8 (72.7%)	35 ± 8	0 (0%)	?	n/a	1.3 (0.7–2.5)^v^	~5.6	4.4 ± 16	40.2 ± 3.6	29.9 ± 4.0	~26.6%^u^	FPG (mmol/L): ↓ pre: 6.0 ± 0.5, post: 5.3 ± 0.5
														HbA1c: ↓ pre: 5.3 [5.3, 5.4], post: 5.2 [4.9, 5.3]
**CEREBRAL BLOOD FLOW**														
^**15**^ **O‐H**_**2**_ **O**														
Redies, 1989^a^	4	OB‐fast^w^	0 (0%)	38 ± 6.2	0 (0%)	?	n/a	0	0.6–0.8	0.6–0.8	36.2 ± 4.1	?	11.8 ± 1.9%	FPG (mmol/L): ↓ pre: 5.4 ± 1.1, post: 4.1 ± 0.3
Delparigi, 2004^b^	11	post‐OB‐LCD^q^	8 (72.72%)	40 ± 6	0 (0%)	?	n/a	n/a	n/a	n/a	> 35	23.6 ± 1.9	?	?
	23	OB	12 (52.2%)	29 ± 6	0 (0%)	?	n/a	n/a	n/a	n/a	n/a	39.6 ± 3.8	n/a	n/a
	21	NWC	10 (47.6%)	33 ± 9	0 (0%)	?	n/a	n/a	n/a	n/a	n/a	22.8 ± 2.1	n/a	n/a
Delparigi, 2007^b^	9	post‐OB‐LCD^q^	9 (100%)	38.0 ± 6.5	0 (0%)	?	n/a	n/a	n/a	n/a	> 35	~23.2	?	?
	20	OB	20 (100%)	31.3 ± 8.6	0 (0%)	?	n/a	n/a	n/a	n/a	n/a	~32.0	n/a	
Le, 2007^b^	8	post‐OB‐LCD^q^	8 (100%)	39 ± 7	0 (0%)	8 (100%)	n/a	n/a	n/a	n/a	> 35	? (65 ± 6 kg)	?	?
	9	OB	9 (100%)	31 ± 8	0 (0%)	9 (100%)	n/a	n/a	n/a	n/a	n/a	? (113 ± 16 kg)	n/a	n/a
	10	NWC	10 (100%)	33 ± 10	0 (0%)	10 (100%)	n/a	n/a	n/a	n/a	n/a	? (61 ± 7 kg)	n/a	n/a
**ASL**														
Almby, 2021	11	RYGB	8 (72.7%)	35 ± 8	0 (0%)	?	n/a	1.3 (0.7–2.5)^v^	~5.6	4.4 ± 16	40.2 ± 3.6	29.9 ± 4.0	~26.6%^u^	FPG (mmol/L): ↓ pre: 6.0 ± 0.5, post: 5.3 ± 0.5
														HbA1c: ↓ pre: 5.3 [5.3, 5.4], post: 5.2 [4.9, 5.3]
**FATTY ACID METABOLISM**														
^**18**^ **F‐FTHA and** ^**11**^ **C‐palmitate**														
Karmi, 2010	16 (overall 23)^k^	MS‐VLCD^r^	11 (68.8%), overall 15 (65.2%)^k^	43 ± 7^k^	? but 100% MS	?	n/a	?	?	1.4 (plus 1 week isocaloric diet)	34.0 ± 3.9	30.2 ± 3.9	~11.1%^u^	FPG (mmol/L): ↓ pre‐VLCD: 10.0 ± 0.6, post‐VLCD: 5.7 ± 0.5
	7	NOC	0 (0%)	42 ± 11	0 (0%)	?	None	n/a	n/a	n/a	26.8 ± 2.5	n/a	n/a	
Rebelos, 2020	21 (overall 24)^l^ (? RYGB, ? VSG)	RYGB/VSG	21 (100%), overall 24 (100%)	43 ± 10	9 (37.5%) T2DM, 4 (16.7%) IGT, 1 (4.2%) IFG	?	n/a	>1	>7	6	41.1 ± 4.2	31.8 ± 4.2	~22.6%^t^ (26 ± 8 kg)	PG (mmol/L): → pre‐RYGB/VSG: 5.7 ± 1.0, post‐RYGB/VSG: 5.3 ± 0.8
	14	NOC	14 (100%)	45 ± 12	0 (0%)	?	None	n/a	n/a	n/a	22.6 ± 2.8	n/a	n/a	HbA1c (%): ↓ pre‐RYGB/VSG: 6.0 ± 0.7, post‐RYGB/VSG: 5.4 ± 0.4

Abbreviations: ?, unknown; →, no change; ↑, increase; ↓, decrease; ^11^C‐DASB, ^11^C‐3‐amino‐4‐(2‐dimethylaminomethyl‐phenylsulfanyl)‐benzonitrile; ^11^C‐MRB, ^11^C‐methylreboxetine; ^123^I‐FP‐CIT, ^123^I‐N‐ω‐fluoropropyl‐2β‐carbomethoxy‐3β‐(4‐iodophenyl)nortropane; ^123^I‐IBZM, ^123^I‐iodobenzamide; ^15^O‐H_2_O, ^15^O‐water; ^18^F‐FDG, ^18^F‐fluorodeoxyglucose; ^18^F‐FTHA, ^18^F‐fluoro‐6‐thia‐heptadecanoic acid; ASL, arterial spin labeling; BMI, body mass index; BR, breakfast; CHO, carbohydrate; D, dinner; DAT, dopamine transporter; FPG, fasting plasma glucose (to convert mmol/L to mg/dL multiply by 18); HbA1c, glycated hemoglobin; IFG, impaired fasting glucose; IGT, impaired glucose tolerance; IQR, interquartile range; IR, insulin resistance; LCD, low‐calorie diet; MS, metabolic syndrome; n/a, not applicable; NOC, non‐obese control; NT, no treatment; NWC, normal weight control (lean); OB, obesity; OW, overweight; PG, plasma glucose; REE, resting energy expenditure; RYGB, Roux‐en‐Y gastric bypass; SD, standard deviation; SERT, serotonin transporter; SPECT, single‐photon emission computerized tomography; T2DM, type 2 diabetes mellitus; VLCD, very low‐calorie diet; VSG, vertical sleeve gastrectomy; YFAS, Yale Food Addiction Scale.

^a^ Same datasets.

^b^ Overlapping dataset.

^c^ Same dataset and tracer (SERT binding at 2 h, DAT binding at 3 h.

^d^ For *n* = 14 overall (includes *n* = 3 without SPECT scan).

^e^ For *n* = 12 overall (includes *n* = 3 without SPECT scan).

^f^ For *n* = 10 overall (includes *n* = 1 excluded from analysis as lost >10% weight).

^g^ For *n* = 7 overall (includes *n* = 1 with only baseline but no post‐VLCD PET scan).

^h^ *n* = 20 baseline, *n* = 16 at 6 months, *n* = 17 at 2 years, *n* = 13 at 3 years.

^i^ For *n* = 22 overall (includes *n* = 5 with only baseline but no post‐RYGB PET scan).

^j^ Higher number at baseline only, lower number post‐LCD.

^k^ For *n* = 23 overall (including *n* = 7 with only baseline and without post‐VLCD PET scan).

^l^ For *n* = 24 overall (including *n* = 3 with only baseline and without post‐RYGB/VSG PET scan).

^m^ 800 kcal per day.

^n^ 50% of total energy requirements (calculated from 1.33 × REE using indirect calorimetry) with 35% at lunch, and either 50% at breakfast, 15% at dinner (LCD‐BR) or 15% at breakfast, 50% at dinner (LCD‐D).

^o^ 800 kcal per day as total meal replacement.

^p^ 1600 kcal/day (30% fat, 50% CHO, 20% protein).

^q^ With diet and exercise BMI fallen from >35 to ≤ 25 kg/m^2^ and weight stable ≥ 3 months.

^r^ 550 kcal per day meal replacement (7% fat, 51% CHO, 42% protein).

^s^ For *n* = 21 at baseline, *n* = 14 post‐intervention.

^t^ Estimated from change in average BMI.

^u^ Estimated from change in average weight.

^v^ For *n* = 18 at baseline.

^w^ Fasted for 3 weeks.

### 3.4 Study protocols and analysis

Study protocols and PET/SPECT protocols and analysis are summarized in Tables S1–S3.

A complete description of study protocols is available in Data S1 Results: 3.4.1. Nutritional status, 3.4.2. Menstrual cycle, 3.4.3. Mood assessment, 3.4.4. PET paradigm and stimulus type, and 3.4.5. PET/SPECT analysis methodology.

Quality of data and risk of bias is summarized in Table S4 and described in Data S1 Results: 3.4.6. Quality of data.

### 3.5 PET/SPECT study findings

Study findings are summarized in Table S5. A complete description of study findings is available in Data S1 Results: 3.5.1. Dopamine neurotransmitter system, 3.5.2. Serotonin neurotransmitter system, 3.5.3. Opioid neurotransmitter system, 3.5.4. Noradrenaline neurotransmitter system, 3.5.5. Regional cerebral blood flow, 3.5.6. Brain glucose uptake, and 3.5.7. Brain fatty acid uptake.

### 3.6 Correlations

Association of PET/SPECT findings with clinical outcomes are summarized in Table S6 and described in Data S1 Results: 3.6.1. Clinical outcomes.

Behavioral measures and their association with PET/SPECT findings are summarized in Tables S7 and S8, and described in Data S1 Results: 3.6.2. Behavioral outcomes and 3.6.3. Mood assessment.

Blood mechanistic measures and the association with PET/SPECT findings are summarized in Tables S9 and S10, and described in Data S1 Results: 3.6.4. Mechanistic outcomes.

## 4 DISCUSSION

This literature review of PET/SPECT studies examining neurotransmitter systems and rCBF and metabolite uptake in surgical and non‐pharmacological weight loss has revealed the difficulties in drawing definitive conclusions as to their effects on brain function and their potential contributions to or consequence of weight loss and changes in appetite and eating behavior. This results from the following factors:

- Limited number of studies examining each neurotransmitter or metabolite system or rCBF, and within neurotransmitter studies the use of different tracers, as well as limited number of participants within each study.
- Variability in type of bariatric surgery used and often combination of multiple types of surgery in single studies.
- Methodological heterogeneity across studies including participant characteristics (age, sex, ethnicity, presence of type 2 diabetes mellitus [T2DM]), timing after intervention, degree of weight loss, nutritional status at scanning session, and statistical analysis.
- Lack of inclusion of appropriate dietary control interventions, for example VLCD or even LCD, in the same study to control for weight loss and reduced energy intake after bariatric surgery.
- Uncommon examination of associations of changes in PET/SPECT outcomes after intervention with clinical outcomes such as weight loss or improvements in glycemic control, changes in measures of eating behavior, or potential mechanistic mediators (e.g., appetitive gut hormones).
- Uncommon inclusion in studies of confounds that may affect the interpretation of PET/SPECT findings such as phase of menstrual cycle, use of psychotropic medications, or improvements in mood.

### 4.1 Dopamine system

Dopamine plays a major role in motivation, reward, and prediction of reward.^54^ Dopamine influences food intake via the mesolimbic circuitry (projections from the ventral tegmental area to regions including the ventral and dorsal striatum) by modulating appetitive motivational processes.^55^, ^56^ Dopaminergic neurotransmission is mediated by five distinct receptor subtypes, which are classified into two main classes of receptors termed D1‐like (D1 and D5) and D2‐like (D2, D3, and D4).^54^ The D2‐like receptors have been associated with feeding and addictive behaviors in human and animal studies.^57^, ^58^, ^59^

Although one small study (*n* = 5) found an *increase* in striatal ^11^C‐raclopride binding potential (BP) at 4–6 weeks after RYGB surgery following ~13% weight loss in the majority of women, no formal statistics was performed,^32^ while no changes were seen in the striatum (or elsewhere in brain) in a larger study (n = 16) of older women at 6 months after RYGB/VSG surgery despite 23% weight loss.^33^ Similarly, no change was observed in striatal ^123^I‐iodobenzamide (^123^I‐IBZM) BP 6 weeks post‐RYGB surgery after average 14kg weight loss,^51^ suggesting that different results are unrelated to temporary early *increases* after surgery or differences in degree of weight loss. However, another study showed an increase in ^123^I‐IBZM BP in striatum and caudate (with trend in putamen) at average 3.1 years after RYGB surgery after 31% weight loss.^52^

By contrast, another small study (*n* = 5) found a *decrease* in ^18^F‐fallypride BP in caudate at ~7 weeks after RYGB/VSG surgery with average ~12% weight loss.^34^ There was a similar trend for a *decrease* in ^18^F‐fallypride BP in caudate, putamen, and nucleus accumbens after 7–10 days of VLCD with average ~3% weight loss in a larger study (*n* = 15),^35^ suggesting that these changes may be because of weight loss or reduced energy intake rather than being specific to bariatric surgery.

To interpret these changes in dopamine 2 and 3 receptors (DRD2/3) receptor availability after weight loss needs an understanding of the effects of obesity or higher BMI itself on DRD2/3 receptor availability. In those interventional studies that examined influence of obesity at baseline, there was no difference in striatal ^11^C‐raclopride BP between participants without obesity/normal weight controls and pre‐operative group with obesity,^32^, ^33^ nor any correlation of striatal ^123^I‐IBZM binding with BMI in pre‐operative group with obesity.^51^, ^52^

However, in other studies, correlations between DRD2/3 receptor availability and BMI or obesity have been highly inconsistent, likely related to (i) multiple different tracers with variable characteristics, (ii) neuroanatomical localization of BP differences, (iii) severity of obesity (with some reviews suggesting inverted U‐shape relationship), (iv) potential differential effects of tonic and phasic dopamine release, and (v) variable sample sizes.^58^, ^60^, ^61^

Higher BMI has been associated with decreased DRD2/3 receptor availability in the ventromedial striatum using ^18^F‐fallypride,^59^ in striatum using ^11^C‐raclopride^62^; in dorsal caudate using 6‐^18^F‐fluoro‐L‐m‐tyrosine^63^; and in ventral striatum, putamen and caudate using 6‐^18^F‐fluoro‐L‐3,4‐dihydroxyphenylalanine.^64^ By contrast, higher BMI has been associated with higher ^18^F‐fallypride BP in the dorsal and lateral striatum^59^; in caudate^65^; in midbrain, putamen, and ventral striatum,^66^ and higher N‐methyl benperidol BP in caudate.^67^ Greater reduction in BMI was positively associated with decrease ^123^I‐N‐ω‐fluoropropyl‐2β‐carbomethoxy‐3β‐(4‐iodophenyl) nortropane, (^123^I‐FP‐CIT) BP over 24 months in caudate and putamen.^68^ No association of BMI has been found with DRD2/3 availability in striatum using N‐methyl benperidol tracer.^67^

^11^C‐4‐propyl‐9‐hydroxynaphthoxazine (^11^C‐PHNO) is more highly selective for DRD3 over DRD2 receptors, and results have differed from the other DRD2/3 tracers. In the same study of participants without obesity (BMI 18.6–27.8 kg/m^2^), BMI was positively correlated with ^11^C‐PHNO BP in ventral striatum (but not caudate or putamen) but not in any striatal region with ^11^C‐raclopride.^69^ Higher BMI (range from 20.8 to 36.5 kg/m2) has also been associated with higher ^11^C‐PHNO BP in the dorsal striatum,^70^ and across those with normal weight, overweight, and obesity in substantia nigra/ventral tegmental area, ventral striatum, and pallidum.^71^ To our knowledge no studies have examined the effects of bariatric surgery or dietary weight loss on ^11^C‐PHNO BP.

Furthermore, ^18^F‐fallypride is not as easily displaced by endogenous dopamine compared to ^11^C‐raclopride and ^123^I‐IBZM tracer and so is less sensitive to changes in endogenous dopamine release.^72^, ^73^, ^74^, ^75^, ^76^ Furthermore, DRD2/3 receptors exist in either high‐ or low‐affinity states with respect to agonists, and while agonist tracers (^11^C‐PHNO, (‐)‐N‐[^11^C]propyl‐norapomorphine (^11^C‐NPA), (R)‐2‐^11^CH3O‐N‐n‐propylnorapomorphine (^11^C‐MNPA)) bind preferentially to the high‐affinity state, antagonists (^11^C‐raclopride, ^11^C‐N‐methylspiperone, ^11^C‐FLB‐457, ^18^F‐fallypride, ^123^I‐IBZM and ^123^I‐epidepride) do not distinguish between the two states.^77^

When looking at voxel‐based analysis rather than averaging BP across striatal brain regions, positive correlations of BMI were found with ^18^F‐fallypride BP in the dorsolateral striatum including caudate and putamen, and negative correlations in the ventromedial striatum, in lean/patients with obesity.^59^

Interpreting changes in baseline ^11^C‐raclopride, ^123^I‐IBZM, and ^18^F‐fallypride BP after weight loss interventions is also difficult because it is assessing post‐synaptic (and potentially also pre‐synaptic auto‐receptors) DA receptor availability rather than the flux through the dopaminergic system. A recent review suggested that the relationship between obesity and DRD2/3 availability can be best described by a nonlinear relationship,^75^ where tracer BP reflects changes in both receptor density and endogenous dopamine levels. The nonlinear relationship may be the result of an increase in tonic dopamine (sustained) levels, accompanied by a decrease in phasic dopamine (momentary) release in moderate obesity which may induce a transient, compensatory upregulation of striatal DRD2/3, resulting in a higher tracer BP in moderate obesity. However, with further progression of obesity (BMI > 40 kg/m^2^), the lower tracer BP may reflect primarily a downregulation of DRD2/3, which can be compensatory to long‐term high tonic dopamine exposure.^78^

The obesity intervention studies using DRD2/3 tracers examined alterations in tonic dopamine, measured during the fasting or pre‐meal state without any active interventions such as presentation of food stimuli or acute food ingestion. Physiologically, dopamine is released in the striatum from midbrain neurons in response to stimuli in a phasic manner. Indeed, greater post‐prandial decreases in striatal ^11^C‐raclopride BP, indicating greater endogenous dopamine release, have been associated with greater pleasantness of the food eaten in adults without obesity.^76^ To our knowledge, there are no published studies of the effects of bariatric surgery or weight loss on post‐prandial endogenous dopamine release.

No association between BMI and striatal dopamine transporter (DAT) availability was found using ^123^I‐FP‐CIT,^79^ whereas a negative association was observed in obesity using (–)‐2‐β‐Carbomethoxy‐3‐β‐(4‐fluorophenyl)tropane (β‐CFT, WIN 35,428) (^3^H‐WIN35,428) tracer^80^ and in participants without obesity (BMI 18–30 kg/m^2^) using TRODAT‐1 tracer.^81^

One study examined the effect of LCD‐induced weight loss on striatal DAT using ^123^I‐FP‐CIT, but this has not been examined after bariatric surgery. Although there was no overall change in striatal ^123^I‐FP‐CIT binding after 1 month LCD following 6–7% weight loss, the timing of the LCD meals over the day (50% of energy intake at breakfast vs. supper) did produce differential effects on striatal ^123^I‐FP‐CIT binding, suggesting the effect of meal timing on weight maintenance after hypocaloric diets.^53^

A further limitation of these obesity interventional studies using tracers targeting the dopamine system is the inclusion of only females, limiting generalization of the results to both sexes.^82^, ^83^

### 4.2 Serotonin system

Serotonin plays an integral role in maintaining energy homeostasis, controlling eating behavior, suppressing appetite, and promoting energy expenditure.^75^, ^84^ Serotonin (5‐HT) receptors are classified into seven types, 5‐HT_1_ through 5‐HT_7_ with each type having subtypes (A, B, etc.). The brain distribution of these receptors is not homogeneous nor identical. Brainstem serotonin neurons send ascending projections that terminate in a defined and organized manner in cortical, limbic, midbrain, and hindbrain regions, with brain regions expressing multiple serotonin receptors in a receptor subtype‐specific fashion.^75^, ^84^

The serotonin system has provided a viable target for weight control.^85^ Serotonin 5‐HT_1B_ and 5‐HT_2C_ receptors have been specifically recognized as mediators of serotonin‐induced reductions in appetite.^85^ Systemic serotonin administration decreases food intake in humans,^86^ and there is an important role for the anorexigenic hypothalamic serotonin 2C receptor (5‐HT_2C_R).^87^ A number of serotonergic drugs, including selective serotonin reuptake inhibitors, dexfenfluramine, and 5‐HT_2C_R agonists, have been shown to attenuate rodent body weight gain. This effect is strongly associated with marked hypophagia and is probably mediated by the hypothalamic melanocortin system.^88^ However, there are inconsistencies in the effect of those drugs on humans.^89^, ^90^, ^91^, ^92^, ^93^ Additionally, sibutramine, dexfenfluramine, fluoxetine, and the 5‐HT_2C_R agonist chlorophenylpiperazine have all been shown to modify appetite in both lean and patients with obesity, resulting in reduced caloric intake.^85^ A new generation of 5‐HT_2C_R selective agonists have been developed such as lorcaserin which helped patients with overweight or obesity to lose weight and maintain weight loss.^85^ In addition, hypothalamic serotonin 2A receptor (5‐HT_2A_R) might have a role in the control of feeding and energy homeostasis. Positive correlations were found between BMI and 5‐HT_2A_R binding using ^18^F‐altanserin tracer in different cortical regions.^94^, ^95^ Individuals with obesity had significantly higher neocortical 5‐HT_2A_R binding relative to lean individuals.^37^ On the other hand, serotonin receptor (SERT) binding was negatively correlated to BMI in cortical and subcortical regions using ^11^C‐3‐amino‐4‐(2‐dimethylaminomethyl‐phenylsulfanyl)‐benzonitrile (^11^C‐DASB) PET tracer.^96^

In the only study of RYGB surgery, there was no effect on ^18^F‐altanserin BP (targeting 5‐HT_2A_R) despite average 25.8% weight loss.^37^ This was despite there being an overall increase in neocortical (averaged across orbitofrontal, medial inferior frontal, superior frontal, medial inferior and superior temporal, sensorimotor, parietal and occipital cortices) ^18^F‐altanserin BP in obesity (both pre‐ and post‐RYGB surgery) than normal weight participants, and a positive correlation with BMI across participants without and with obesity. In agreement with these findings, two other studies found a positive correlation between BMI (across range from participants without and with obesity) and ^18^F‐altanserin binding in the neocortex (averaged across eight cortical anatomical regions of interest (aROIs): orbitofrontal, medial inferior frontal, superior frontal, superior temporal, medial inferior temporal, sensory‐motor, parietal, and occipital cortices), and also individually in the above aROIs, as well as insula, hippocampus, anterior cingulate cortex and posterior cingulate cortex, in one study,^95^ and in the other study in the superior temporal, medial inferior temporal, dorsolateral prefontal, and sensory‐motor cortical aROIs (but not cerebellum, amygdala/hippocampus, pons, orbitofrontal cortex, ventrolateral frontal cortex, anterior cingulate gyrus, thalamus, caudate, putamen/pallidum, insula, superior medial frontal cortex, occipital cortex, or parietal cortex).^94^

The lack of any reduction in ^18^F‐altanserin BP after weight loss from RYGB surgery suggests persistence of alterations in the serotonin system in obesity, perhaps consistent with lower intra‐synaptic serotonin concentrations. However, because there are no reported studies of weight loss induced by a dietary intervention on ^18^F‐altanserin BP, it is unclear if this is a general lack of effect from weight loss or whether RYGB surgery actually increases ^18^F‐altanserin BP.

The ^11^C‐Cimbi PET tracer is also available to target 5‐HT_2A_R in humans, but no studies could be found assessing influence of BMI, obesity, or interventions on its binding.^97^, ^98^

In rats with diet‐induced obesity from high fat diet, RYGB surgery decreased ^3^H‐MDL100907 binding by autoradiography (targeting 5‐HT_2A_R) in the nucleus accumbens (but not cortex, caudate/putamen, hippocampus, or hypothalamus) compared with sham operated rats, but no changes were seen in SERT (using (S)‐[*N*‐methyl‐^3^H]citalopram) or 5‐HT_4_R (using ^3^H‐SB207145) binding restriction.^99^

Unfortunately, there are no specific tracers for the anorexigenic 5‐HT_2C_R. Radioligands for the other serotonin 1A and 1B (5‐HT_1A/B_R) and 4 (5‐HT_4_R) receptors have been validated in humans, but there are no reported studies of their use in surgical or dietary weight loss interventions.

One study showed no effect of RYGB surgery on ^11^C‐DASB BP (targeting SERT) averaged across caudate, putamen, and thalamus, despite 25.8% weight loss.^37^ In agreement with this, studies have found no difference in ^11^C‐DASB BP between participants with and without obesity,^100^ and with other tracers targeting SERT, no correlation between BMI and ^123^I‐labeled 2β‐carboxymethoxy‐3β‐(4‐iodophenyl)tropane (^123^I‐nor‐β‐CIT) BP across participants without and with obesity,^96^, ^101^ nor correlation of BMI with midbrain/cerebellum ratio of ^123^I‐(2‐((2‐([dimethylamino]methyl)phenyl)thio)‐5‐iodophenylamine (^123^I‐ADAM) BP across participants without obesity and participants with severe obesity,^102^ indicating that SERT is unaltered in obesity.

However, although LCD producing 6.5% weight loss had no overall effect on ^123^I‐FP‐CIT BP in thalamus and hypothalamus, an increase in tracer BP in thalamus was seen when 50% of energy was consumed in breakfast (vs. supper), suggesting that thalamus SERT may be affected by timing of dietary patterns but not weight loss per se.^53^

### 4.3 Opioid system

There are three main families of opioid receptors (μ, ĸ, and δ) of which μ‐opioid receptors (MOR) are most strongly implicated in reward processing. The endogenous opioid system and MOR influence food and energy balance, particularly by modulating consummatory behavior beyond changes in appetite.^103^, ^104^, ^105^ Additionally, the opioid system is involved in the regulation of affective and stress responses and is therefore positioned as a common mediator that underlies the interface of food intake, hedonic response, and emotional regulation.^106^, ^107^, ^108^ Administration of MOR antagonists to animals reduces food intake and body weight in rodent models,^109^, ^110^, ^111^, ^112^ while MOR agonists increase food intake.^113^, ^114^ In humans, pharmacological studies of high affinity but non‐selective MOR antagonists such as naloxone, naltrexone and nalmefene found decreases in short‐term food intake but no effects on hunger in participants with normal weight.^115^, ^116^, ^117^ Recently, studies using a selective MOR antagonist GSK1521498 showed reductions in hedonic responses to sweetened dairy products and reduced energy intake, particularly of high‐fat foods during ad libitum buffet meals in obesity with binge eating disorder,^118^, ^119^ and reduced attentional bias for food cues on the visual dot probe task.^120^

Two studies observed an increase in ^11^C‐carfentanil BP after both RYGB/VSG surgical and VLCD dietary weight loss interventions in ventral striatum, thalamus, and orbitofrontal cortex, suggesting this is because of weight loss itself rather than changes in gut‐brain axis from surgery.^33^, ^36^ After bariatric surgery but not dietary interventions there were also increases in ^11^C‐carfentanil BP in amygdala, dorsal caudate, insula, putamen, and anterior, middle and posterior cingulate cortex,^33^ whereas an increase in ^11^C‐carfentanil BP in temporal pole was observed after dietary but not surgical interventions.^36^

The anatomical differences in the increases in ^11^C‐carfentanil BP between surgical and dietary interventions may be a result of the greater weight loss in the former (23.3% vs. 16.1%, respectively) as well as the time since start of intervention (6.0 vs. 3.7 months, respectively). Moreover, the surgical intervention study was much larger than the dietary study (16 vs. 7 participants), and there were differences in participant sex (all female in surgical, all male in dietary study), prevalence of T2DM (38% vs 0%), and nutritional state (fed in surgical, fasted in dietary study) which further impairs the comparison between these two studies.^36^

These results suggest that weight loss by surgical or dietary interventions is normalizing the lower ^11^C‐carfentanil BP seen in obesity (pre‐intervention vs. participants without obesity) in ventral striatum, dorsal caudate, putamen, thalamus, amygdala, insula, posterior cingulate cortex and orbitofrontal cortex (average and individual regions of interests [ROIs]),^33^ thalamus, amygdala, temporal pole, and prefrontal cortex.^36^ These cross‐sectional findings in obesity are supported by others that have found lower ^11^C‐carfentanil BP in ventral striatum, dorsal caudate, putamen, insula, amygdala, thalamus, orbitofrontal cortex, and posterior cingulate cortex.^61^

There are no PET studies investigate ĸ‐ and δ‐opioid receptors in human obesity or interventions. Preliminary data from transgenic knockout models suggest that mice lacking some of these receptors are resistant to high fat diet‐induced obesity, suggesting a role of these receptors in controlling energy metabolism.^121^, ^122^ Moreover, the κ‐specific antagonist norbinaltorphimine showed robust reductions in the intake of palatable diets high in fat or sucrose.^123^, ^124^, ^125^, ^126^

### 4.4 Noradrenaline system

The main source of noradrenergic neurons is the midbrain locus coeruleus projecting to many areas in the central nervous system, and they influence a broad range of physiological and behavioral functions, including arousal, memory, attention, and mood.^127^, ^128^, ^129^ Noradrenaline also plays an important role in energy balance.^128^, ^129^ In rodent studies, exogenous noradrenaline can elicit or reduce feeding, depending on the site of infusion (lateral hypothalamus stimulates feeding; perifornical hypothalamus inhibits feeding; lesions of the ascending ventral noradrenergic bundle increases food intake and produces obesity, whereas interruption of projections of the dorsal noradrenergic bundle lowers body weight) and the relative balance of post‐synaptic α2‐adrenoceptors (stimulate food intake) and α1‐adrenoceptors (inhibit food intake).^130^, ^131^ These two adrenoceptor subtypes are localized in the hypothalamic paraventricular nucleus and appear to be organized in an antagonistic fashion.^132^

The noradrenaline transporters (NAT) take up synaptically released noradrenaline and thus serves as a primary mechanism for inactivation of noradrenergic signaling.^133^, ^134^, ^135^

In the only study, there was no effect of LCD intervention on ^11^C‐methylreboxetine (^11^C‐MRB) BP (targeting NAT) after 3.7% weight loss over 6 months.^47^ However, the weight loss was minimal, and the participants still had obesity after the intervention with average BMI 41.0 kg/m^2^. However, greater weight loss after LCD was associated with a greater increase in ^11^C‐MRB BP in the insula and hippocampus, but the role of noradrenergic signaling on energy balance in these brain regions is unclear. Furthermore, lower ^11^C‐MRB BP at baseline was associated with greater weight loss after LCD in insula and hippocampus, and also putamen, midbrain, and dorsolateral prefrontal cortex.^47^

A recent study that investigated the effect of RYGB surgery on NAT observed a higher ^11^C‐MRB BP in hypothalamus at baseline was associated with greater weight loss 6 months post‐RYGB surgery, a brain region responsible for appetite control and homeostasis. Moreover, reductions in BMI after RYGB surgery was associated with reductions in NAT availability in the dorsolateral prefrontal cortex and a general tendency towards reduced NAT throughout the brain.^136^ However, these preliminary findings need confirmation with larger cohorts.

While this direction of change in ^11^C‐MRB BP with weight loss has been supported by cross‐sectional studies in obesity, the exact brain regions involved have differed: (i) in lean‐to‐ participants with severe obesity, higher BMI was associated with lower ^11^C‐MRB BP in the hypothalamus,^137^ whereas (ii) participants with class I obesity (mean BMI 34.7 kg/m^2^) had lower ^11^C‐MRB BP in the thalamus but not hypothalamus compared to lean participants.^138^ However, these results have not been replicated in more severe class II and class III obesity (BMI > 35 kg/m^2^).^139^, ^140^

It therefore remains uncertain if impaired NAT availability is a definite feature of obesity and if it is playing any pathogenic role in overeating behavior. A number of anti‐obesity drugs have targeted the noradrenaline system though rarely used clinically because of adverse effect profiles particularly due to peripheral monoamine release such as increased heart rate and blood pressure. Their mechanisms of action are complex though, because they often affect multiple monoamine neurotransmitter systems, for example, sibutramine reduces reuptake of noradrenaline and also serotonin and dopamine; phentermine and amphetamine stimulate monoamine release from neurons via trace‐amine associated receptor 1 (TAAR1) receptor including noradrenaline and, to a lesser extent, serotonin and dopamine.^128^ The potential reduced NAT uptake in obesity and its increase with dietary weight loss could therefore represent a counter‐regulatory response to obesity rather than a pathogenic cause.

### 4.5 Fatty acid uptake

The hypothalamic metabolism of fatty acids can modify feeding behavior and has been proposed to function as a biochemical sensor for nutrient availability that in turn exerts negative feedback on nutrient intake.^87^, ^141^, ^142^ The mechanisms by which hypothalamic long‐chain fatty acid (acyl‐CoAs) concentrations can be increased are enhanced esterification of circulating or central nervous system lipids^143^, ^144^ and/or by the local inhibition of lipid oxidation.^145^ These interventions also result in marked inhibition of feeding behavior in pre‐clinical studies.^146^, ^147^, ^148^, ^149^ In animal studies, saturated fats disturb melanocortin signaling of hypothalamic neuronal subgroups pivotal to energy balance.^150^, ^151^, ^152^ Moreover, hypothalamic injury can occur in response to increased dietary fat very early (1–3 days) even before the development of obesity in rodents,^153^ and the normalization of hypothalamic lipid sensing has been linked to normalization of energy and glucose homeostasis in rats.^154^

In addition, free fatty acids induce insulin and leptin resistance which may cause neuronal damage through inflammation including the hypothalamus and so further affect control of energy balance.^151^, ^155^, ^156^ Hypothalamic overexpression of a constitutively active IKKβ isoform (which is activated by saturated fatty acids and oxidative stress) can reduce both insulin and leptin signaling^151^; conversely, intracerebroventricular administration of an IKKβ inhibitor reverses high fat diet‐induced hypothalamic insulin resistance,^157^ and neuron‐specific deletion of IKKβ maintains leptin and insulin sensitivity in high fat diet fed mice.^151^ These control processes are difficult to examine in humans in vivo, and so most data in this regard have only been demonstrated in animals.^146^, ^148^, ^158^ One key unresolved question regarding the effect of fatty acids in the brain is the nature of the cell types and if there are other brain regions involved in the response.

Both PET studies of dietary and surgical weight loss interventions showed higher brain ^18^F‐fluoro‐6‐thia‐heptadecanoic acid (^18^F‐FTHA) BP (which measures total FA uptake and is found mostly in triglycerides in brain lipids) globally and in cortical regions in obesity (pre‐intervention vs. participants without obesity),^46^, ^49^ as well in sub‐cortical and hypothalamus in one study.^46^ However, only the dietary intervention study observed a reversal with weight loss with a decrease in ^18^F‐FTHA BP globally and regionally in cortical, sub‐cortical, and hypothalamus 1.5 months after VLCD with 11.1% weight loss.^46^ However, ^18^F‐FTHA BP was unchanged 6 months post‐RYGB/VSG surgery in cortical regions despite greater 22.6% weight loss to a similar BMI to the post‐VLCD study.^49^ Unfortunately, this surgical study did not include the hypothalamus as a region of interest. Instead, they measured the ratio of hypothalamic‐to‐amygdala signal intensity (using fluid‐attenuated inversion recovery, FLAIR‐MRI) which has been previously shown to reflect hypothalamic inflammation,^153^ but this did not differ between participants with obesity and controls at baseline nor change after surgery.^49^ The authors mentioned this may be a result of methodological limitations because of slice thickness of 5 mm.

Thus, these differences between the two studies in changes in ^18^F‐FTHA BP in cortical regions are unlikely to be explained by magnitude of weight loss, but there could be adaptation to weight loss over time, or else surgical intervention increases ^18^F‐FTHA BP through uncertain mechanisms. The authors hypothesized that surgical stress may be a factor, but this is unlikely to be important at 6 months post‐surgery.^49^ Moreover, there were differences between these studies in sex ratio (all female in surgical study, 68.8% female in dietary study) and baseline BMI (average 41 kg/m^2^ in surgical study, 34 kg/m^2^ in dietary study), which further impairs the direct comparison between the studies if being female or having more severe obesity reduces reversibility with weight loss, though no evidence is yet available for this.^46^, ^49^

^11^C‐palmitate measures non‐oxidative fatty acid uptake and is found mostly in phospholipids in brain lipids, with only trace amounts in triglycerides and fatty acids. Interestingly, ^11^C‐palmitate BP did not change after weight loss from VLCD dietary intervention, suggesting that the greater ^18^F‐FTHA BP in obesity, and decrease in ^18^F‐FTHA BP after VLCD, is primarily because of decreases in oxidative fatty acids, which are those associated with inflammation and neuronal damage.^46^

### 4.6 Regional cerebral blood flow

Regional cerebral blood flow can be used to assess local neuronal activity at rest and/or in response to interventions because of the neurovascular coupling that results in local vasodilation. rCBF can be measured by PET imaging with ^15^O‐water (^15^O‐H_2_O)^12^ and by magnetic resonance imaging using arterial spin labeling (ASL).^159^

One small longitudinal study with only males with obesity (*n* = 4) showed no change in rCBF using ^15^O‐H_2_O PET averaged across the whole brain after 3 weeks of total fasting.^43^ Only one larger study (*n* = 11) assessed the effect of RYGB surgery on rCBF, in this case using ASL.^50^ After RYGB, there was increased rCBF in the whole brain, white and gray matter, and individually within caudate, putamen, pallidum, thalamus, amygdala, hippocampus, hypothalamus, frontal, parietal, temporal and occipital lobes, and cerebellum, during normoglycemia and in most of these brain regions during hypoglycemia.^50^ This suggests differential global changes in neuronal activity after weight loss from RYGB surgery than extreme dietary restriction. However, interpretation of these findings is complicated by (i) neither study including normal weight participants (unclear what direction of change would be expected to normalize obesity‐associated changes in rCBF), (ii) global effects raise the possibility of non‐specific effects after RYGB surgery, (iii) prolonged fasting was a dietary intervention that is an unusual treatment, (iv) samples sizes were small, and (v) these two studies used different methods to assess rCBF.

Furthermore, another longitudinal study using ASL found no change in rCBF at 6 months after RYGB surgery versus pre‐operatively (*n* = 9) nor any difference in rCBF at baseline compared to controls without obesity (*n* = 8), in any regional brain network defined using resting state functional MRI (dorsal default mode, ventral default mode, auditory, basal ganglia, left or right executive control, language, precuneus, sensorimotor network, primary visual, visuospatial, higher visual, anterior salience, and posterior salience networks).^160^

Three cross‐sectional studies used ^15^O‐H_2_O PET to compare successful dieters with non‐dieters with obesity (and sometimes also those who never had obesity) to measure rCBF responses to taste or intake of a liquid meal (Ensure) but with overlapping datasets.^44^, ^45^, ^48^ However, none of these studies just compared rCBF between groups when fasted.

In the insula (a brain region that includes the taste cortex), increase in rCBF after taste (but not after food intake) relative to fasting was higher in both non‐dieters with obesity and successful dieters (but similar between groups) than those who have never had obesity, suggesting a persistence of potentially pathogenic abnormality from obesity even after dietary‐induced weight loss.^44^, ^45^, ^48^

Few studies have examined the effects of obesity surgery on brain responses to sweet taste using fMRI.^161^, ^162^ Interestingly, one study found a reduction in blood oxygen level dependent (BOLD) signal to chocolate milk taste (sweet, high fat) in the insula (which includes gustatory cortex) after RYGB surgery.^161^ Furthermore, this was attenuated by acute administration of the glucagon‐like peptide‐1 (GLP‐1) analog Exendin(9–39), indicating a potential role for the increased plasma GLP‐1 after RYGB in these changes of sweet/fat taste responsivity.^163^, ^164^

In the hippocampus and parahippocampal gyrus (regions involved in memory and learning), rCBF after food intake decreased more in both non‐dieters with obesity and successful dieters (but similar between groups) than those who have never had obesity, again suggesting a persistence of response from obesity even after dietary‐induced weight loss,^44^ but this was only replicated for non‐dieters with obesity in a reanalysis of this study.^48^

By contrast, in the amygdala and posterior cingulate cortex, a greater increase in rCBF after food intake was seen in non‐dieters with obesity than both successful dieters and participants who never had obesity, suggesting a reversible consequence of obesity that normalizes after weight loss.^44^ However, these findings were not replicated in the other two studies.^45^, ^48^

By contrast, more consistent results were found in the dorsal and dorsolateral pre‐frontal cortex (a region involved in top‐down inhibitory control^165^), with a greater decrease in rCBF after food intake in non‐dieters with obesity than both successful dieters and participants who never had obesity.^45^, ^48^ This is supported by other studies finding lower rCBF in those with compared to without obesity using ^15^O‐H_2_O PET during fed state^166^, ^167^ and during response to a liquid meal.^168^, ^169^, ^170^ Reduced prefrontal cortex function in obesity when fasted or after food intake may contribute to a lack of inhibition of overeating in obesity,^171^ and impaired cessation of a feeding episode, as the dorsal prefrontal cortex has efferent inhibitory projections to the central orexigenic system.^172^ Indeed, impairments of prefrontal cortex function have been associated with eating dysregulation and weight gain in many human lesion studies such as dementia.^173^, ^174^, ^175^

Although not always replicated or regions were not re‐examined, rCBF after food intake (vs. fasted) was greater in putamen, and lower in orbitofrontal cortex and occipital lobe in successful dieters (but not those who never had obesity) than non‐dieters with obesity,^45^, ^48^ whereas rCBF after food intake was greater in cerebellum, and lower in STG and MTG, in successful dieters than those who never had obesity.^45^, ^48^

Several factors may contribute to differences between these ^15^O‐H_2_O PET studies that investigate response to food, including sex ratio (both sexes,^44^ only females^45^, ^48^), different pre‐processing steps,^45^, ^48^ and statistical analyses (single‐level, fixed‐effect analysis^44^; second‐level, random‐effects re‐analysis^48^, ^166^).

### 4.7 Brain glucose uptake

The brain uses glucose as a primary fuel for energy generation. Glucose enters the brain by facilitated diffusion across the blood–brain barrier. BGU can be used to assess local neuronal activity by PET imaging with ^18^F‐FDG tracer,^176^ though glucose transport might also be altered during changes in non‐neuronal glucose uptake (e.g. astrocytes, glia cells)^177^ and non‐specific changes in cerebral glucose metabolism and/or insulin resistance and plasma glucose concentrations.^178^, ^179^ Several studies investigated BGU post‐bariatric surgery^38^, ^39^, ^40^, ^41^, ^50^ or post‐dietary intervention,^42^, ^43^ but the findings are sometimes difficult to compare because of methodological differences, especially around nutritional and metabolic state.

In one cross‐sectional study, BGU was measured in response to food intake post‐RYGB surgery compared with adults with and without obesity,^38^ whereas in longitudinal studies, one study measured BGU in response to hyperinsulinemic normoglycemic or hypoglycemic clamps post‐RYGB surgery,^50^ and two studies during hyperinsulinemia normoglycemic clamp post‐RYGB/VSG surgery.^39^, ^41^ During hyperinsulinemia normoglycemic clamps, there was a decrease in whole brain BGU post‐RYGB surgery^50^ and post‐RYGB/VSG surgery in one of the two studies which included patients with T2DM,^39^ but not the other without patients with T2DM, despite similar weight loss.^41^ This may be consistent with the reductions in insulin resistance seen after bariatric surgery, though none of these studies correlated changes in BGU with changes in insulin resistance.

A cross‐sectional study of response to food intake post‐RYGB surgery found greater increase in BGU in the hypothalamus, pituitary, and medial orbitofrontal cortex compared with controls with and without obesity, and greater decrease in BGU in dorsolateral prefrontal cortex and default mode network (posterior cingulate gyrus, precuneus cortex, cuneus, angular gyrus, superior temporal gyrus posterior, middle temporal gyrus posterior, occipital pole, and parietal lobule) compared with controls with and without obesity.^38^ Surprisingly, these changes post‐RYGB surgery appeared to be largely independent of gut hormone release as they persisted after administration of the somatostatin analog Octreotide that suppresses satiety gut hormones such as peptide YY (PYY) and GLP‐1.

One longitudinal study of RYGB surgery examined BGU without a hyperinsulinemic clamp but did not report the nutritional state of participants.^40^ The two dietary intervention studies only measured BGU during the fasting state^42^, ^43^; however, one was after 3 weeks of total fasting without any task,^43^ whereas the other was while viewing high‐energy, palatable food pictures.^42^

No studies were found investigating the effect of VSG alone (always combined with RYGB surgery as one group), gastric banding, or biliopancreatic diversion for obesity on neurotransmitter systems or brain metabolism, nor the effects of any obesity surgery on the noradrenaline system.

### 4.8 Correlations of PET/SPECT findings with clinical outcomes

Results from the studies examining associations of PET/SPECT findings (at baseline or their change post‐intervention) with clinical outcomes did not offer reproducible evidence that their changes predict weight loss or improvements in glucose metabolism because of the paucity of studies with each intervention, tracer and neurotransmitter system, and lack of consistency between the overall effects of intervention on neuroimaging outcomes and correlations.^35^, ^36^, ^37^, ^39^, ^42^, ^47^, ^49^, ^52^

For example, looking at *baseline* PET results correlating with weight loss, (i) higher BP in neocortex for 5HT_2A_R but not serotonin transporter was correlated with greater weight loss post‐RYGB surgery^37^; (ii) a greater post‐prandial increase in MOR availability in temporal pole was correlated with less weight loss after VLCD intervention^36^; (iii) no correlation was observed between baseline BGU and weight loss post‐RYGB/VSG surgery^39^; while (iv) higher BP for NAT in putamen, hippocampus, midbrain, insula, and dorsolateral prefrontal cortex was correlated with less weight loss post‐LCD intervention.^47^

When looking at correlation of *changes* in PET/SPECT findings with weight loss: (i) despite no overall changes in BP after the intervention, a smaller increase in neocortex 5HT_2A_R availability, and in caudate, putamen, and thalamus for serotonin transporter, was correlated with greater weight loss post‐RYGB^37^; (ii) no correlations between loss of weight nor fat mass and change in DRD2/3 receptor availability were seen post‐RYGB despite changes in BP being seen after surgery^52^; (iii) a greater increase in NAT in hippocampus and insula was associated with greater weight loss post‐LCD, despite no overall change in transporter post‐dietary intervention^47^; while (iv) changes in BGU did not correlate with loss of weight or fat post‐LCD.^42^

When looking at correlation of *baseline* PET/SPECT findings with changes in glycemic control, two studies of RYGB/VSG surgery for obesity (with 32–38% having T2DM) found that: (i) higher whole brain BGU (during insulin stimulation) was correlated with less improvement in fasting plasma glucose (FPG) at 3 years, perhaps indicative of better insulin sensitivity at baseline with a floor effect^39^; and similarly (ii) higher whole brain free fatty acid (FFA) uptake was correlated with less improvement in FPG at 2 years.^49^

When looking at correlation of *changes* in PET/SPECT findings with changes in glycemic control, (i) there was no correlation between increase in DRD2/3 availability (^123^I‐IBZM BP) in striatum with decrease in FPG at 3 years post‐RYGB surgery for obesity (with unknown number having T2DM at baseline)^52^; while (ii) greater reduction in DRD2/3 availability (^18^F‐fallypride BP) in caudate, putamen, and substantial nigra correlated with greater decrease in FPG 10 days post‐VLCD for obesity (only 7% with T2DM).^35^

### 4.9 Correlations of PET/SPECT findings with mechanistic measures

Bariatric surgery involves a profound anatomical change to the gastrointestinal tract, which causes a more rapid delivery of nutrients to the distal small bowel.^3^, ^180^ As a result, after RYGB and VSG surgery, gut adaptation facilitates an exaggerated, early post‐prandial rise in peripheral anorexigenic gut hormones including PYY and GLP‐1, and a reduced post‐weight loss rise in fasting and/or post‐prandial plasma concentrations of the potentially orexigenic stomach‐derived hormone ghrelin, likely as a result of the exclusion of food from the stomach (though the majority of studies have examined total rather than acyl ghrelin), that occurs within days after surgery and persists long term.^3^, ^181^ These appetitive gut hormones have receptors in the peripheral and central nervous systems forming a gut‐brain hormonal axis. Therefore, these obesity surgeries promote weight loss by reducing appetite, partly mediated by changes in appetitive gastrointestinal hormone secretion.^3^, ^5^

Furthermore, the effects of RYGB and VSG surgery on gut hormones are different from the effects of dietary intervention.^181^ Fasting plasma total ghrelin decreased more after RYGB surgery than matched weight loss from VLCD, whereas post‐oral glucose plasma total ghrelin was unchanged after RYGB surgery, but increased after matched weight loss from diet alone.^182^, ^183^ Post‐oral glucose plasma GLP‐1 increased after RYGB surgery for obesity with T2DM, but not after matched weight loss from LCD.^184^ In addition, despite similar weight loss, fasting and post‐prandial acyl ghrelin may decrease more after VSG than RYGB surgery, while post‐prandial plasma PYY_3‐36_ and active GLP‐1 may increase more after RYGB than VSG surgery.^185^

Observations of differences in PET/SPECT outcomes between surgical and dietary interventions implicate some of these mechanistic changes in gut anatomy–physiology after surgery compared with dietary intervention,^46^, ^49^ as opposed to similar effects for surgical and non‐surgical interventions that implicate mechanisms related to weight loss itself or perhaps psychological changes attempting to inhibit excess energy intake.^33^, ^36^

However, when looking at roles for specific mechanisms, a limited number of studies have assessed correlations between PET/SPECT findings and potential mediators, again meaning that definitive conclusions cannot be made. No correlations were seen among the following: (i) changes in fasting total ghrelin (overall no change) or decrease in serum insulin and increase in striatum DRD2/3 availability (^123^I‐IBZM BP) post‐RYGB surgery;^52^ (ii) changes in fasting acyl ghrelin (overall no change) or decrease in fasting serum insulin and decreases in DRD2/3 availability (^18^F‐fallypride) in ventral striatum, caudate, and putamen post‐VLCD;^35^ and (iii) increase in post‐prandial plasma GLP‐1 (400 kcal) and changes in SERT (average caudate, putamen, and thalamus) or 5‐HT_2A_R (neocortex) availability (^11^C‐DASB or ^18^F‐altanserin BP) post‐RYGB surgery.^37^

Acute administration of the somatostatin analog Octreotide to patients after RYGB surgery to suppress anorexigenic gut hormones GLP‐1 and PYY (with co‐administration of insulin to avoid hyperglycemia) had no effect on BGU (fed vs. fasted) in sub‐callosal gyrus, hypothalamus, insula, precuneus, cuneus, posterior cingulate cortex, dorsolateral prefrontal cortex, orbitofrontal cortex, frontal operculum, angular gurus, parietal lobule, superior temporal gyrus, middle temporal gyrus, occipital lobe, and lingual gyrus.^38^ This was despite these regions being those showing differences in post‐prandial BGU in patients post‐RYGB surgery compared to participants with obesity or normal weight controls, suggesting that the exaggerated post‐prandial GLP‐1 and PYY responses after RYGB surgery were not responsible for changes in regional BGU, though sample size was small for the post‐RYGB group (*n* = 9). This is in contrast to an fMRI study of food cue reactivity, where acute suppression of post‐prandial plasma GLP‐1 and PYY with Octreotide increased food picture appeal and cue reactivity across nucleus accumbens, anterior insula, amygdala, and caudate post‐RYGB surgery (but not gastric banding), while the greater the suppression of plasma PYY and GLP‐1, the greater the increase in food cue reactivity across both post‐surgical groups.^186^

### 4.10 Correlations of PET/SPECT findings with behavioral measures

Similarly, very few studies have assessed correlations between PET/SPECT findings and changes in eating behavior precluding any definitive conclusions of brain changes with behaviors leading to weight loss: (i) the decrease in state (but not trait) food craving was positively correlated to the increase in striatal DRD2/3 availability (^123^I‐IBZM BP) 3 years post‐RYGB^52^; (ii) changes in post‐prandial 5‐HT_2A_R and SERT availability did not correlate with increased post‐prandial satiety post‐RYGB surgery, though this is unsurprising as overall there was no change in the PET outcomes.^37^

None of the studies included in this systematic review correlated change in PET measures with change in food liking or wanting score, changing in taste function, nausea, dumping syndrome, or food aversion.

### 4.11 Correlations of PET/SPECT measures with mood

Most longitudinal studies did not measure changes in mood,^35^, ^38^, ^39^, ^40^, ^41^, ^42^, ^43^, ^44^, ^45^, ^46^, ^48^, ^49^, ^50^, ^51^, ^52^, ^53^ and some found no change in mood post‐RYGB or VSG surgery^33^, ^34^, ^37^ or LCD^47^ or VLCD,^36^ whereas one study showed lower depression post‐RYGB surgery that was associated with a reduction in DRD2/3 availability (^11^C‐raclopride BP) across ventral striatum, caudate, and putamen, though no direct correlation was performed.^32^ Improvements in mood are often seen after bariatric surgery,^187^, ^188^ and so may be a cofounding factor when interpreting PET findings. For example, depression is associated with higher DRD2/3 availability (^11^C‐raclopride BP) in putamen region.^189^

### 4.12 Interactions between neurotransmitter systems

Furthermore, published studies have generally examined neurotransmitter systems and brain regions in isolation and have not examined how the neurotransmitter systems interact with each other and how they work on a systemic level such as in the brain reward system. Only two longitudinal studies included multiple tracers looking at neurotransmitter systems in the same participants, but none looked at correlations between changes in the different tracer BPs as a result of the intervention. There were increases in ^11^C‐carfentanil BP (MOR) in ventral and dorsal striatum, but no changes in ^11^C‐raclopride BP (DRD2/3) in these regions, in a longitudinal study of RYGB/VSG surgery,^33^ that normalized the reductions in ^11^C‐carfentanil BP seen in obesity (vs. without obesity), with no effect of obesity for ^11^C‐raclopride BP.^33^, ^61^ Examining dopamine and serotonin transporter (both FP‐CIT) in a longitudinal study of 4 weeks LCD found no changes in former and changes in serotonin transporter BP in thalamus, the direction of which depended on distribution of energy intake over the day.^53^

Interaction of dopaminergic/noradrenergic systems with opioid and serotonin systems is demonstrated from PET studies of effects of oral administration of amphetamine, which increases dopaminergic and noradrenergic systems (via dopamine and noradrenaline transporter inhibition, vesicular monoamine transporter 2 [VMAT‐2] inhibition, and monoamine oxidase activity inhibition).^97^, ^190^, ^191^ Amphetamine administration released endogenous beta‐endorphin and serotonin as measured by reductions in BP for ^11^C‐carfentanil (MOR agonist) in putamen, caudate, nucleus accumbens, frontal cortex, anterior cingulate cortex, insula, and thalamus,^190^, ^191^ and by reductions in ^11^C‐CIMBI‐36 (5HT‐2A receptor agonist) in frontal, parietal, temporal, and occipital cortex.^97^ However, while blunting of these effects of amphetamine have been reported in gambling disorder and abstinent alcohol dependence,^192^, ^193^ and depression,^194^ to our knowledge they have not been studied in obesity or following its treatment.

Positive correlations between DRD2 and MOR availability using ^11^C‐raclopride and ^11^C‐carfentanil BP were reported in the ventral striatum and caudate but not in the putamen in lean participants, and in severe obesity the correlation in the ventral striatum was attenuated, suggesting aberrant mesolimbic dopamine–opiate interaction in obesity.^195^ However, it has not yet been reported whether surgical or dietary interventions for obesity normalize this correlation in the ventral striatum.

The poor temporal resolution of PET/SPECT imaging precludes examination of temporal interactions of dynamic changes in neurotransmitter systems between brain regions that is better explored using resting state or task‐related functional connectivity, a topic outside the scope of this review, that has been examined in several fMRI studies.^16^, ^196^, ^197^, ^198^, ^199^, ^200^, ^201^, ^202^, ^203^

### 4.13 Limitations

Although it was hoped to conduct a meta‐analysis, this was not possible because of several limitations from the available studies: (i) combined groups composed of patients who underwent different surgeries which have differing effects on gut anatomy and physiology, (ii) different times since surgery or start of dietary intervention, (iii) small number of included manuscripts for each brain neurotransmitters system or metabolite, let alone the specific PET/SPECT tracer used, (iv) different nutritional and metabolic states used between studies, (v) different ROIs used in particular studies further decreasing the number of studies that could be included in a meta‐analysis, and (vi) very few studies reported spatial co‐ordinates from whole brain analysis precluding combination of results using an ALE analysis (using GingerALE software, http://brainmap.org). In addition, this systematic review did not focus on the different analytical models used in quantification in PET/SPECT data.

### 4.14 Recommendations

There are notable gaps in the literature. We offer the following recommendations to further accelerate the field's understanding of the effect of obesity surgery on neurotransmitter systems and brain metabolism and to determine the potential of these surgeries for the clinical treatment of obesity:

- Enrolment of larger sample sizes with greater representation across age and sex, particularly studies involving young adults and males.
- Subgrouping according to the type of the surgery and classification of participants according to BMI.
- Including a control group for effects of weight loss or dietary/psychological advice.
- Examine the effect of VSG surgery, because 20% of the bariatric surgery studies included in this systematic review had mixed groups post‐RYGB/VSG, and no studies examined VSG alone, nor included gastric banding or biliopancreatic diversion surgery.
- Careful consideration regarding the control groups used (e.g., controlling for BMI, T2DM, age, mood, and medication).
- Simultaneous assessment of multiple biomarkers (e.g., mechanistic outcome) to determine the additive value of each marker in the clinical assessment of brain function.
- Address mediators of the effect of the intervention on brain function (e.g., hormonal change and behavior change).
- Correlate change in PET/SPECT measures with change in food liking or wanting score, change in taste function, nausea, dumping syndrome or food aversion.
- Although it would be best to have a double‐blind, randomized control study design in studies involving surgical procedures, this is difficult because of logistical and ethical issues.
- Some of the reviewed studies only included one sampling time point (if any) for gastrointestinal hormones, usually in the fasted state. It is of interest to determine how these appetitive hormones are affected in the postprandial state. Therefore, future studies should sample before and after a meal to capture the gastrointestinal hormone response profile.
- Reporting data using whole brain analysis or/and standardization of ROIs so meta‐analysis can be easily performed.
- Assessment of interactions between neurotransmitter systems and their association with changes in functional MRI measures, for example, food cue reactivity or resting state functional connectivity, aided by dual PET/MRI scanners now being available.

### 4.15 Conclusions

There is an increase in MOR BP post‐RYGB/VSG surgery and VLCD intervention, suggesting changes in the opioid system may be secondary to weight loss or reduced energy intake rather than changes in gut‐brain axis from surgery. It also suggests that weight loss normalizes the lower ^11^C‐carfentanil BP seen in obesity. BGU both globally and regionally usually decreased after bariatric surgery, and was also seen with LCD and prolonged fasting, again suggesting the effects are because of weight loss itself or reduced energy intake. The findings are sometimes difficult to compare because of methodological differences, especially around nutritional and metabolic state.

Results from the studies examining associations of PET/SPECT findings with clinical outcomes did not offer reproducible evidence that their changes predict weight loss or improvements in glucose metabolism because of the paucity of studies with each intervention, tracer, and neurotransmitter system, and lack of consistency between overall effects of intervention on neuroimaging outcomes and correlations. A limited number of studies have assessed correlations between PET/SPECT findings and potential mediators or behavioral outcomes, again meaning that definitive conclusions cannot be made. Most longitudinal studies did not measure changes in mood which may be a cofounding factor when interpreting PET/SPECT findings. None of the studies included in this systematic review correlated changes in PET/SPECT measures with changes in food liking or wanting score, taste function, nausea, dumping syndrome or food aversion.

The small number of studies with each tracer and lack of control groups made definitive conclusions challenging. Variability in methodology used, duration since intervention, amount of weight loss, nutritional status, methods of statistical analysis, small sample size, predominantly females included, and the use of combined surgical groups also limit conclusions. These limitations need to be addressed in future studies examining the effects of different bariatric surgeries in order to fully understand the role for changes in neurotransmitter systems or brain metabolism involved in changing eating behavior. This will help us understand the mechanisms that cause weight loss after surgical interventions and in return help tailor treatments for the patient and identify potential therapeutic targets for non‐surgical weight loss in obesity.

## AUTHOR CONTRIBUTIONS

Conceptualization: Alhanouf S. Al‐Alsheikh, Alexander D. Miras, Anthony P. Goldstone; methodology: Alhanouf S. Al‐Alsheikh, Anthony P. Goldstone; validation: Alhanouf S. Al‐Alsheikh, Shahd Alabdulkader, Anthony P. Goldstone; investigation: Alhanouf S. Al‐Alsheikh; resources: Alhanouf S. Al‐Alsheikh, Shahd Alabdulkader, Anthony P. Goldstone; data curation: Anthony P. Goldstone; writing—original draft preparation: Alhanouf S. Al‐Alsheikh, Anthony P. Goldstone; writing: Alhanouf S. Al‐Alsheikh, Anthony P. Goldstone; review and editing: Alhanouf S. Al‐Alsheikh, Shahd Alabdulkader, Alexander D. Miras, Anthony P. Goldstone; visualization: Alhanouf S. Al‐Alsheikh, Anthony P. Goldstone; supervision: Alexander D. Miras, Anthony P. Goldstone; project administration: Anthony P. Goldstone. All authors have read and agreed to the published version of the manuscript.

## CONFLICT OF INTEREST STATEMENT

No conflict of interest statement.

## Supporting information


**Data S1.** Supporting Information

**Table S1.** Study protocols.
**Table S2.** PET/SPECT protocols.
**Table S3.** PET/SPECT analysis.
**Table S4.** Quality assessment.
**Table S5.** PET/SPECT results.
**Table S6.** PET/SPECT association with clinical outcomes.
**Table S7.** Behavioral measures.
**Table S8.** PET/SPECT association with behavioral measures.
**Table S9.** Blood mechanistic measures.
**Table S10.** PET/SPECT association with blood mechanistic measures.
