# Supplementary material for: Effects of bariatric surgery and dietary interventions for obesity on brain neurotransmitter systems and metabolism: A systematic review of positron emission tomography (PET) and single‐photon emission computed tomography (SPECT) studies
Source: Obes Rev. 2023 Sep 12;24(11):e13620. doi: 10.1111/obr.13620 (PMC10909448; doi:10.1111/obr.13620)
Supplement: Supplementary file 1 — Data S1. Supporting Information [file OBR-24-e13620-s001.docx]

**SUPPLEMENTARY INFORMATION**

**Effects of Bariatric Surgery and Dietary Interventions for Overweight and Obesity on the Brain:
a Systematic Review of Positron Emission Tomography (PET) and Single-Photon Emission Computed Tomography (SPECT) Studies**

Alhanouf S. Al-Alsheikh ^a, b^, Shahd Alabdulkader ^c^, Alexander D. Miras ^a,d^, Anthony P. Goldstone ^e^

^a^ Department of Metabolism, Digestion and Reproduction, Imperial College London, Hammersmith Hospital, London W12 0NN, UK

^b^ Department of Community Health Sciences, College of Applied Medical Sciences, King Saud University, Riyadh 11451, Saudi Arabia

^c^ Department of Health Sciences, College of Health and Rehabilitation Sciences, Princess Nourah Bint Abdulrahman University, Riyadh 84428, Saudi Arabia

^d^ Ulster University, School of Medicine, Faculty of Life & Health Sciences, Londonderry, Northern Ireland, BT48 7JL, UK

^e^ PsychoNeuroEndocrinology Research Group, Division of Psychiatry, Department of Brain Sciences, Imperial College London, Hammersmith Hospital, London W12 0NN, UK

Email: tony.goldstone@imperial.ac.uk

**CONTENTS**

**SUPPLEMENTARY METHODS**

2.2. Database Search

*2.2.1. Keywords/terms used*

2.3. Data extraction

**SUPPLEMENTARY RESULTS**

3.2. Study Summaries

*3.2.1. PET/SPECT tracers*

*3.2.2. Country*

3.3. Demographic Data

*3.3.1. Participant characteristics*

*3.3.2. Time since intervention*

*3.3.3. Weight loss ranges*

*3.3.4. Control groups*

3.4. Study Protocols and Analysis

*3.4.1. Nutritional status*

*3.4.2. Menstrual cycle*

*3.4.3. Mood assessment*

*3.4.4. PET paradigm and stimulus type*

*3.4.5. PET/SPECT analysis methodology*

*3.4.6. Quality of data*

3.5. PET/SPECT Study Findings

*3.5.1. Dopamine neurotransmitter system*

*3.5.2. Serotonin neurotransmitter system*

*3.5.3. Opioid neurotransmitter system*

*3.5.4. Noradrenaline neurotransmitter system*

*3.5.5. Regional cerebral blood flow*

*3.5.6. Brain glucose uptake*

*3.5.7. Brain fatty acid uptake*

3.6. Correlations

*3.6.1. Clinical outcomes*

*3.6.2. Behavioural outcomes*

*3.6.3. Mood assessment*

*3.6.4. Mechanistic outcomes*

**SUPPLEMENTARY REFERENCES**

**SUPPLEMENTARY TABLES**

Table S1 Study protocols

Table S2 PET/SPECT protocols

Table S3 PET/SPECT analysis

Table S4 Quality assessment

Table S5 PET/SPECT results

Table S6 PET/SPECT association with clinical outcomes

Table S7 Behavioural measures

Table S8 PET/SPECT association with behavioural measures

Table S9 Blood mechanistic measures

Table S10 PET/SPECT association with blood mechanistic measures

**SUPPLEMENTARY METHODS**

**2.2. Database Search**

***2.2.1. Keywords/terms used***

The keywords used in the database searches were: (obesity OR overweight OR diabetes) AND (brain) AND [(surgery) OR (weight loss) OR (fat loss) OR (weight reduction) OR (weight maintenance) OR (gastric bypass) OR (RYGB) OR (sleeve gastrectomy) OR (VSG) OR (LVSG) OR (gastric sleeve) OR (gastric band) OR (gastric banding) OR (gastroplasty) OR (stomach balloon) OR (gastric balloon) OR (biliary pancreatic diversion) OR (diet) OR (lifestyle modification) OR (behavioural therapy) OR (psychological therapy)] AND [(PET) OR (positron emission tomography) OR (SPECT) OR (single photon emission computed tomography) OR (fluorodeoxyglucose) OR (FDG) OR (H2O) OR (raclopride) OR (fallypride) OR (PHNO) OR (DAT) OR (dopamine transporter) OR (carfentanil) OR (serotonin) OR (5HT) OR (5-HT) OR (opioid) OR (noradrenaline) OR (dopamine) OR (fatty acid) OR (cerebral blood flow)]. Once the database searches were accomplished, the papers listed were analysed based on the exclusion and inclusion criteria by examination of the titles, abstracts, and methods used.

**2.3. Data Extraction**

The core data generated from each article were:

1. Study summary (authors name, publication year, journal, country, tracer used, target system in the brain, study design, if including bariatric surgery or non-surgical dietary intervention or control group, type of intervention, task, paradigm nutritional state (fed vs. fasted) or other state intervention (e.g. acute drug vs. placebo), inclusion of clinical, mechanistic or behaviour outcomes and correlation with PET/SPECT findings, exclusion criteria: use of psychotropic medication).
2. Demographic characteristics and clinical outcomes (sample size, sex ratio, age, T2DM, ethnicity, control intervention, time scan pre- and post-intervention, time between scans, BMI at baseline and post-intervention, weight loss, improvements in glycaemic control).
3. Study protocol (state manipulation, nutritional state, whether controlled for feeding/manipulation order effects, type of meal and macronutrient composition; time since last meal (defined as fasted ≥ 8h, pre-meal >2 to <8 h, fed ≤ 2h); whether controlled for menstrual cycle or mood).
4. PET/SPECT protocol (radioligand name, PET or SPECT, task during PET/SPECT scan (if any), task paradigm, duration of scan, reference region).
5. PET/SPECT analysis (software, analysis methodology, statistical threshold; correction for multiple comparison, covariates).
6. PET/SPECT results (relevant contrasts (e.g. post- vs. pre-intervention, post-surgery vs. unoperated, fasted vs. fed etc.), analysis method (whole brain, small volume correction, anatomical or functional ROIs), reported by brain region.
7. Eating behaviour and other psychological measures (appetite ratings, food wanting/liking, food intake, eating behaviour questionnaires, other cognitive outcomes, mood).
8. Mechanistic measures (appetitive gut hormones (e.g. ghrelin, GLP-1, PYY), leptin, insulin, insulin resistance, nausea ratings, symptoms of dumping syndrome).

**SUPPLEMENTARY RESULTS**

**3.2. Study Summaries**

***3.2.1. PET/SPECT tracers***

Radioactive tracers used to investigate neurotransmitter systems illustrated in Figure 2 in the main paper. One or more tracers were used for each neurotransmitter system: (i) for dopamine system, three different tracers were used for D2RD2/3 (11-raclopride, ^123^l-IBZM and 18F-fallypride) and one tracer was used for DAT (^123^l-FP-CIT); (ii) for serotonin system, one tracer was used for 5-HT_2A_R (18F-altanserin), and two tracers were used for serotonin transporter (^11^C-DASB, ^123^I-FP-CIT); (iii) only one tracer was used for noradrenaline (^11^C-MRB) and (iv) one tracer for MOR (11C-carfentanil).

Radioactive tracers used to investigate brain metabolism illustrated in Figure 3 in the main paper. For assessment of brain metabolism: (i) Fluorodeoxyglucose (^18^FDG) tracer was used for measuring brain glucose uptake (BGU); (ii) ^18^F- FTHA was used to measure total fatty acid uptake and 11C-palmitate to measure non-oxidised fatty acid uptake; (iii) ^15^O-H_2_O labelled water was used for rCBF. In addition, although it is a not a PET tracer technique, one ^18^FDG study also used the MRI technique of ASL which also measures rCBF.

***3.2.2. Country***

Seven of the 22 studies were carried out in the USA (31.8%) (Delparigi et al. 2004; DelParigi et al. 2007; Le et al. 2007; Dunn et al. 2010; Steele et al. 2010; Burghardt et al. 2015; Dunn et al. 2017), five in Finland (22.7%) (Karmi et al. 2010; Tuulari et al. 2013; Karlsson et al. 2016; Rebelos et al. 2019; Rebelos et al. 2020), three in the Netherlands (13.6%) (de Weijer et al. 2014; van der Zwaal et al. 2016; Versteeg et al. 2017) and one study (4.5%) was conducted in each of the following countries: Denmark (Haahr et al. 2015), United Kingdom (Hunt et al. 2016), Brazil (Marques et al. 2014), Italy (Guzzardi et al. 2018), Canada (Redies et al. 1989), Sweden (Almby et al. 2021) and Germany (Vettermann et al. 2018). Five publications (22.7%) contained overlapping datasets from two protocols (de Weijer et al. 2014; van der Zwaal et al. 2016) and (Delparigi et al. 2004; DelParigi et al. 2007; Le et al. 2007), while two publications (9.1%) (Redies et al. 1989; Karlsson et al. 2016) contained the same dataset, leaving 13 completely independent datasets.

**3.3. Demographic Data**

Demographic data for individual studies is given in Table 2 in the main paper.

***3.3.1. Participant characteristics***

With regard to PET sample size in the intervention group, nine studies (40.9%) included less than or equal to 10 participants (Redies et al. 1989; DelParigi et al. 2007; Le et al. 2007; Dunn et al. 2010; Steele et al. 2010; Burghardt et al. 2015; Hunt et al. 2016; Versteeg et al. 2017; Vettermann et al. 2018); eight studies (36.4%) included between 11 and 20 participants (Delparigi et al. 2004; de Weijer et al. 2014; Marques et al. 2014; Karlsson et al. 2016; van der Zwaal et al. 2016; Dunn et al. 2017; Guzzardi et al. 2018; Almby et al. 2021); and six studies (27.3%) included over 20 participants (Karmi et al. 2010; Tuulari et al. 2013; Haahr et al. 2015; Guzzardi et al. 2018; Rebelos et al. 2019; Rebelos et al. 2020). Note that the total number of studies here is more than the number of publications as some studies had more than one intervention group.

The total number of baseline participants across all studies was 558, 440 of whom were female (78.9%), with eleven studies including only females (DelParigi et al. 2007; Le et al. 2007; Dunn et al. 2010; Steele et al. 2010; de Weijer et al. 2014; Marques et al. 2014; Karlsson et al. 2016; van der Zwaal et al. 2016; Dunn et al. 2017; Guzzardi et al. 2018; Rebelos et al. 2020), seven with ≥50% females (Delparigi et al. 2004; Karmi et al. 2010; Tuulari et al. 2013; Haahr et al. 2015; Hunt et al. 2016; Rebelos et al. 2019; Almby et al. 2021), one study included 40% female (Vettermann et al. 2018) and three studies including only males (Redies et al. 1989; Burghardt et al. 2015; Versteeg et al. 2017).

Only eight out of 22 studies (36.4%) reported participants’ ethnicity (Le et al. 2007; Dunn et al. 2010; Steele et al. 2010; de Weijer et al. 2014; Hunt et al. 2016; van der Zwaal et al. 2016; Dunn et al. 2017; Vettermann et al. 2018) and out of these 163 participants, 132 (81.0%) were white Caucasian.

Eleven studies (50%) were conducted exclusively on patients without type 2 diabetes mellitus (T2DM) (Redies et al. 1989; Delparigi et al. 2004; DelParigi et al. 2007; Le et al. 2007; Dunn et al. 2010; Steele et al. 2010; Marques et al. 2014; Versteeg et al. 2017; Guzzardi et al. 2018; Vettermann et al. 2018; Almby et al. 2021), while five studies (22.7%) included some participants with T2DM (Tuulari et al. 2013; Karlsson et al. 2016; Dunn et al. 2017; Rebelos et al. 2019; Rebelos et al. 2020), and six studies (27.3%) did not report the prevalence of T2DM (Karmi et al. 2010; de Weijer et al. 2014; Burghardt et al. 2015; Haahr et al. 2015; Hunt et al. 2016; van der Zwaal et al. 2016), although one of these only included participants with metabolic syndrome (Karmi et al. 2010).

***3.3.2. Time since intervention***

In longitudinal studies, the time of PET/SPECT scanning post-intervention varied greatly from as early as 8 or 10 days (Dunn et al. 2017) to 3.1 years (van der Zwaal et al. 2016). Seven studies conducted PET/SPECT scans from 3 weeks to 3 months post-intervention (Redies et al. 1989; Dunn et al. 2010; Karmi et al. 2010; Steele et al. 2010; de Weijer et al. 2014; Versteeg et al. 2017; Guzzardi et al. 2018), two studies from 3 to 6 months after intervention (Burghardt et al. 2015; Almby et al. 2021), six studies at 6 months after intervention (Delparigi et al. 2004; Tuulari et al. 2013; Marques et al. 2014; Karlsson et al. 2016; Rebelos et al. 2019; Rebelos et al. 2020), and one study 8 months post-intervention (Haahr et al. 2015).

In cross-sectional studies, one study carried out PET scanning 1.5 years after surgery (Hunt et al. 2016), while the other three studies did not clearly report the time after intervention (Delparigi et al. 2004; DelParigi et al. 2007; Le et al. 2007).

***3.3.3. Weight loss ranges***

The mean percentage weight loss in surgery intervention studies was variable in part related to variable duration of follow-up, with average of 11-20% (Dunn et al. 2010; Steele et al. 2010), over 20% (Tuulari et al. 2013; Marques et al. 2014; Haahr et al. 2015; Hunt et al. 2016; Karlsson et al. 2016; van der Zwaal et al. 2016; Rebelos et al. 2019; Rebelos et al. 2020; Almby et al. 2021), and up to 31% (Hunt et al. 2016; van der Zwaal et al. 2016). Meanwhile in dietary intervention group studies, the average weight loss was generally lower being less than 5% (Dunn et al. 2017; Guzzardi et al. 2018; Vettermann et al. 2018), 6-10% (Versteeg et al. 2017), or 11-20% (Redies et al. 1989; Karmi et al. 2010; Burghardt et al. 2015). The individual results are summarised in Table 2 in the main paper.

***3.3.4. Control groups***

Fifteen of the studies included a non-interventional control group, either normal weight participants (BMI <25 kg/m^2^) (Delparigi et al. 2004; Le et al. 2007; Steele et al. 2010; Marques et al. 2014; Burghardt et al. 2015; Haahr et al. 2015; Hunt et al. 2016) or participants without obesity (BMI <30 kg/m^2^) (Karmi et al. 2010; Tuulari et al. 2013; Karlsson et al. 2016; van der Zwaal et al. 2016; Vettermann et al. 2018; Rebelos et al. 2019; Rebelos et al. 2020) or with obesity without an intervention (Delparigi et al. 2004; DelParigi et al. 2007; Le et al. 2007). However, none of the studies compared findings between two different interventions (surgery vs. dietary, or between different surgeries).

**3.4. Study Protocols and Analysis**

Study protocols are summarised in Supplementary Table S1

***3.4.1. Nutritional status***

In eleven studies (50.0%) participants were scanned when fasted: five after “overnight” fasting (de Weijer et al. 2014; van der Zwaal et al. 2016; Versteeg et al. 2017; Rebelos et al. 2019; Almby et al. 2021); two studies, after fasting for 8 hours (Dunn et al. 2010; Dunn et al. 2017); and four after 10-14 hours fasting (Redies et al. 1989; Karmi et al. 2010; Tuulari et al. 2013; Rebelos et al. 2020). One study (4.5%) assessed participants pre-meal (6 hours since last meal) (Guzzardi et al. 2018). In two studies (9.1%), participants were fed (2 hours after meal) (Karlsson et al. 2016) (did not report the time since last meal) (Haahr et al. 2015) and five studies (22.7%) included two nutritional states, fasted (>10 hours) and fed (30 minutes since meal) (Burghardt et al. 2015), fasted (>9 hours) and fed (1 hour since meal) (Hunt et al. 2016), fasted (36 hours) and fed (30 minutes since meal) (Delparigi et al. 2004; DelParigi et al. 2007; Le et al. 2007). Three studies (13.6%) did not report nutritional status (Steele et al. 2010; Marques et al. 2014; Vettermann et al. 2018).

***3.4.2. Menstrual cycle***

Only five studies (22.7%) including pre-menopausal women controlled for stage of the menstrual cycle, with four conducted during the follicular phase (Delparigi et al. 2004; DelParigi et al. 2007; Le et al. 2007; Guzzardi et al. 2018), while one study scanned in the first ten days of menstrual cycle (Hunt et al. 2016).

***3.4.3. Mood assessment***

Only six studies (27.3%) assessed mood and psychological factors as a potential confound for differences in PET/SPECT findings using Beck Depression Inventory-II questionnaire (BDI-II) (Dunn et al. 2010; Steele et al. 2010; Karlsson et al. 2016), state-trait anxiety inventory questionnaire (STAI) (Karlsson et al. 2016), adult temperament questionnaire (ATQ) (Vettermann et al. 2018), positive and negative affect schedule questionnaire (PANAS) (Burghardt et al. 2015), symptom checklist-90-R (SCL-90-R) questionnaire, major depression index and Cohen’s perceived stress (Haahr et al. 2015).

However, only one of these studies observed an improvement in mood 4-6 months post-RYGB surgery (Steele et al. 2010), while no change in mood was observed 1.6 months post-RYGB/VSG (Dunn et al. 2010), 8.2 months post-RYGB (Haahr et al. 2015), 6 months post-LCD (Vettermann et al. 2018), 3.6 months post-VLCD (Burghardt et al. 2015) or 6 months post-VLCD (Karlsson et al. 2016).

***3.4.4. PET paradigm and stimulus type***

Summary of PET/SPECT protocols methodology is given in Supplementary Table S2. Three dietary intervention studies used sensory stimulation inside the PET scanner. One study used ^18^F-FDG to study the effect of LCD used three sensory stimuli involving visual, olfactory and taste during neutral (e.g., landscape) or palatable food cues (e.g., chocolate cake) (Guzzardi et al. 2018). Two studies used rCBF (^15^O-H_2_-O) to investigate the effect of LCD used a gustatory stimulus (after tasting and after consuming a satiating liquid meal) (Delparigi et al. 2004; DelParigi et al. 2007).

***3.4.5. PET/SPECT analysis methodology***

Summary of PET/SPECT analysis methodology is given in Supplementary Table S3. Only one (4.5%) study used whole brain analysis only (Marques et al. 2014); nine studies (40.9%) used a predefined ROIs from previous study (Redies et al. 1989; Dunn et al. 2010; Steele et al. 2010; de Weijer et al. 2014; Haahr et al. 2015; van der Zwaal et al. 2016; Dunn et al. 2017; Guzzardi et al. 2018; Almby et al. 2021); ten studies (45.5%) used both whole brain and ROIs analyses (including on occasion for secondary correlational analyses) (Delparigi et al. 2004; DelParigi et al. 2007; Karmi et al. 2010; Tuulari et al. 2013; Burghardt et al. 2015; Hunt et al. 2016; Karlsson et al. 2016; Vettermann et al. 2018; Rebelos et al. 2019; Rebelos et al. 2020); while one study (4.5%) used whole brain and small volume correction analyses (Le et al. 2007); and one study (4.5%) used both ROIs and small volume correction (for striatum only) analyses (Versteeg et al. 2017).

Only fourteen studies (63.6%) corrected for multiple comparisons in their PET/SPECT analysis of main effects of the intervention (rather than secondary correlations): (i) in whole brain analysis studies (Delparigi et al. 2004; Karmi et al. 2010; Tuulari et al. 2013; Marques et al. 2014; Burghardt et al. 2015; Hunt et al. 2016; Karlsson et al. 2016; Rebelos et al. 2019; Rebelos et al. 2020); (ii) in ROIs studies (Redies et al. 1989; Dunn et al. 2010; Karmi et al. 2010; Karlsson et al. 2016; Guzzardi et al. 2018); and (iii) in small volume correction studies (Delparigi et al. 2004; Le et al. 2007; Versteeg et al. 2017).

Only five studies (22.7%) reported power calculations (de Weijer et al. 2014; Karlsson et al. 2016; Dunn et al. 2017; Versteeg et al. 2017; Rebelos et al. 2019).

Seven studies (31.8%) included covariates in the analysis, adjusting for smoking and medication (antidiabetic, antihypertensive and cholesterol-lowering drugs) with ^11^C-raclopride tracer in RYGB/VSG patients (Karlsson et al. 2016), age and whole brain blood flow with ^15^O-H_2_O tracer after low calorie diet intervention (DelParigi et al. 2007), whole brain blood flow with ^15^O-H_2_O tracer after low calorie diet intervention (Delparigi et al. 2004), age with ^18^F-FDG tracer in RYGB patients (Marques et al. 2014; Hunt et al. 2016), education level with ^18^F-FDG tracer in RYGB patients (Marques et al. 2014), and physical activity with ^18^F-FDG tracer in RYGB/VSG patients (Tuulari et al. 2013), scanner with ^18^F-FTHA and ^11^C-palmitate tracers in VLCD (different PET-scanners were used in this study) (Karmi et al. 2010).

***3.4.6. Quality of data***

It is important to realise that none of the papers identified in this systematic review came from randomised clinical trials but only from observational cross-sectional and longitudinal observational studies. The average score on the NIH Quality Assessment Scale was 69.7% (range 60-80%). Sixteen studies had good quality, six studies had fair quality and no studies had poor quality (Supplementary Table S4).

**3.5. PET/SPECT Study Findings**

***3.5.1. Dopamine neurotransmitter system***

*11C-raclopride*: Two longitudinal studies examined effects of RYGB and/or VSG surgery and comparing with normal-weight participants (BMI 18-25 kg/m^2^) (Steele et al. 2010) or participants without obesity (BMI 18-30 kg/m^2^) (Karlsson et al. 2016). There was an increase in dopamine receptor D2 (DRD2) binding-potential (BP) in the average anatomical region of interests (aROIs) (ventral striatum, anterior/posterior putamen and caudate) 4-6 weeks after RYGB surgery in unknown nutritional state (n=5) (Steele et al. 2010), but no change was observed six months after RYGB/VSG in individual aROIs (ventral striatum, caudate, putamen) when fed (n=16) (Karlsson et al. 2016). However, neither study showed differences in DRD2 BP comparing the pre-operative group with obesity and the control group without obesity (Steele et al. 2010; Karlsson et al. 2016)

*18F-fallypride***:** Two longitudinal studies examined effects at seven weeks after RYGB/VSG surgery (n=5) (Dunn et al. 2010), and ten days after a VLCD intervention (n=5) (Dunn et al. 2017), both done when fasted using aROIs. Both these studies showed a reduction in DRD2/3 BP in the substantia nigra, however a decrease in caudate, medial thalamus, amygdala and hypothalamus was only seen after RYGB/VSG surgery (Dunn et al. 2010), though there as a trend for a decrease after VLCD in the ventral striatum, putamen, caudate and hypothalamus (Dunn et al. 2017).

*^123^I-IBZM***:** Two longitudinal studies examined effects after RYGB surgery when fasted using aROIs. One showed no changes in DRD2/3 BP in caudate, putamen and whole dorsal/ventral striatum at six weeks after RYGB surgery (n=19) (de Weijer et al. 2014), while the other demonstrated an increase in DRD2/3 BP in caudate and whole striatum with a similar trend towards in putamen 3 years after RYGB surgery (n=11) (van der Zwaal et al. 2016). In the latter study, D2/3 BP in the whole striatum post-RYGB was lower than in a group without obesity (van der Zwaal et al. 2016).

*^123^I-FP-CIT:* One longitudinal, dietary intervention study using aROI (striatum) showed no change in DAT BP four weeks after a low-calorie diet (LCD) in combined subgroups (where 50% of energy requirement was consumed at breakfast (n=9) or dinner (n=11) (Versteeg et al. 2017). However, DAT BP in the striatum increased more after LCD in the breakfast group compared to the dinner group. Using small volume correction analysis DAT BP increased in the ventral striatum in the breakfast group and decreased in the dorsal striatum in the dinner group.

***3.5.2. Serotonin neurotransmitter system***

*^123^I FP-CIT:* One longitudinal study showed no differences in extra-striatal aROIs (thalamus, hypothalamus) serotonin transporter BP four weeks LCD in the fasting state in combined subgroups (50% of kCal given at breakfast or dinner) (n=20) (Versteeg et al. 2017). However, when comparing the two groups, serotonin transporter BP increased in the thalamus after LCD in the breakfast group with a decrease in the dinner group.

*^18^F-altanserin:* One longitudinal study showed no change in 5-HT_2A_R BP in the neocortex (volume-weighted average of eight cortical regions: OFC, medial inferior frontal, superior frontal, superior temporal, medial inferior temporal, sensory-motor, parietal and occipital cortices) 8 months after RYGB surgery (n=12) in the fed state using aROIs (Haahr et al. 2015). However, a higher neocortical 5-HT_2A_R BP was observed in both pre- and post-RYGB surgery compared with normal weight participants.

*^11^C-DASB:* The same longitudinal study also used an ^11^C-DASB tracer to examine changes in serotonin transporter BP averaged across caudate, putamen and thalamus aROIs 8 months after RYGB surgery in the fed state (n=13) (Haahr et al. 2015). No changes were observed in BP after surgery, nor where there any differences between pre- or post-RYGB surgery compared with normal weight participants.

***3.5.3. Opioid neurotransmitter system***

*11C-carfentanil:* In one longitudinal study of bariatric surgery in the fed state, there was an increase in MOR 11C-carfentanil BP six months after RYGB/VSG surgery in the following individual aROIs (and averaged across all aROIs) (n=16): ventral striatum, dorsal caudate, putamen, thalamus, amygdala, insula, ACC, medial cingulate cortex, PCC and OFC (Karlsson et al. 2016). Similar increases were seen in these regions using whole brain analysis except for putamen, medial cingulate cortex and PCC. Interestingly, 11C-carfentanil BP was lower pre-RYGB/VSG surgery than a control group without obesity on average across all aROIs and in individual aROIs except ACC and medial cingulate cortex, but no difference when comparing post-RYGB/VSG with controls without obesity. No effect of T2DM diagnosis was seen on 11C-carfentanil BP in any aROIs pre-surgery.

In a second longitudinal study of VLCD in the fasted study, there was an increase in 11C-carfentanil BP in the ventral striatum, thalamus, medial OFC cortex and temporal pole in whole brain analysis, about four months after VLCD (Burghardt et al. 2015). A lower 11C-carfentanil BP in thalamus, amygdala, temporal pole and PFC was observed in before VLCD compared with a group with normal weight, while a lower 11C-carfentanil BP was observed in the frontal pole and temporal pole after VLCD compared with group with normal weight. There was also an increase in 11C-carfentanil BP in a fasted compared with fed state in the ventral striatum and frontal pole in pre-VLCD group; in the ventral striatum, thalamus, amygdala and temporal pole in post-VLCD group; and in the ventral striatum, thalamus, amygdala, frontal pole, medial OFC and temporal pole in group with normal weight.

***3.5.4. Noradrenaline neurotransmitter system***

*^11^C-MRB:* One longitudinal study in demonstrated no change in NAT ^11^C-MRB BP after six months of LCD in unknown nutritional state (n=10) in individual aROIs: ventral striatum, caudate head, putamen, thalamus, amygdala, hippocampus, hypothalamus, locus coeruleus, insula, medial prefrontal cortex, ACC, dlPFC, OFC and midbrain (Vettermann et al. 2018).

***3.5.5. Regional cerebral blood flow***

*^15^O-H_2_O:* One longitudinal study found no change in rCBF averaged across the whole brain after 3 weeks complete fasting except water and electrolyte supplements (only n=4) (Redies et al. 1989).

One cross-sectional study, comparing rCBF between successful dieters (after LCD) (n=11), group with obesity who were not dieting (n=23), and group with normal weight (n=21 (Delparigi et al. 2004) when fasted (36 hours), immediately after consuming a small (2 ml) quantity of liquid HE food (taste) and 30 mins after food intake (fed). Using fROIs analysis (for those regions showing a significant interaction between group and state), there was greater insula rCBF after tasting (vs. fasted) in both the post-LCD and obesity groups compared to the group with normal weight, and a lower PCC rCBF in the group with obesity compared to the normal weight but not post-LCD group. When fed (vs. fasted) there was greater increase in rCBF in amygdala and PCC in both the successful dieters and group with normal weight compared to group with obesity, with no difference between the former groups. When fed (vs. fasted), both successful dieters and group with obesity showed show a greater decrease in hippocampus rCBF than normal-weight participants, with no difference between the former groups.

Another cross-sectional study by the same group demonstrated using whole brain analysis a greater reduction in rCBF in the hippocampus, parahippocampal gyrus and occipital lobe after a 2mL taste of liquid meal following a 36 h fast in successful dieters (n=9) compared with group with obesity who are not dieting (n=20) (DelParigi et al. 2007). However, there was greater increase in rCBF in the putamen, dorsal frontal pole, dorsal prefrontal cortex, and anterior cerebellum, and less increase in rCBF in OFC, after consuming a meal (fed vs. fasted), in successful dieters compared with group with obesity who are not dieting.

In another cross-sectional study comparing fed with fasted states, women with obesity had lower rCBF in left dlPFC and IFG than both women of normal weight and those participants who were formerly had obesity (post-LCD), with no differences between the latter two groups (Le et al. 2007). In addition, rCBF in OFC and occipital lobe when fed (vs. fasted) was lower in group post-LCD compared with group with obesity, while rCBF in superior and left middle temporal gyri was lower post-LCD than in normal weight groups (Le et al. 2007). Furthermore, when fed (vs. fasted), rCBF in dlPFC, IFG, hippocampus and parahippocampal gyrus was lower, and rCBF in ACC, dlPFC and MFG was higher in group with obesity compared to group of normal weight.

*Arterial spin labelling (ASL):* Only one study assessed CBF post-RYGB surgery but using ASL (a functional MRI technique rather than PET imaging using radiolabelled water) (Almby et al. 2021). At average 4 months post-RYGB, rCBF in the fasted state had increased in all brain regions during normoglycemia and in most brain regions during hypoglycaemia (but no regional coordinates given) using hyperinsulinaemic clamps, but no effects of hypoglycaemia itself were seen.

***3.5.6. Brain glucose uptake***

In a longitudinal RYGB study in unknown nutritional state, there was a decrease in BGU using ^18^F-FDG in the uncus, parahippocampal gyrus, PCC, middle temporal lobe, anterior cerebellum, and IFG six months after RYGB in whole brain analysis (n=17) (Marques et al. 2014). Furthermore, compared with normal weight participants, a higher BGU was observed pre-RYGB surgery in PCC and posterior cerebellum, while no differences were observed post-RYGB surgery in any brain regions (Marques et al. 2014).

In another longitudinal RYGB study in fasted state, there was also a decrease in grey matter BGU after RYGB surgery during a hyperinsulinaemic normoglycemic clamp using both whole brain and aROIs analyses (though results for individual brain regions were not reported) (n=11) (Almby et al. 2021). Hyperinsulinaemic hypoglycaemia increased grey matter BGU to a similar degree post-RYGB as pre-RYGB surgery, but there was a greater decrease in BGU in the hypothalamus post-RYGB than pre-RYGB surgery with hypoglycaemia.

Another smaller cross-sectional RYGB study, compared participants on average 18 months post-RYGB (n=9) with unoperated patients with obesity (n=21), and normal-weight (n=12) participants in different nutritional state (fed 400 kcal - vanilla ice cream vs. fasted) in whole brain analysis (Hunt et al. 2016). Participants post-RYGB showed: (i) higher BGU after eating (fed vs. fasted) than participants with obesity or normal weight in ventral cingulate subcallosal gyrus, hypothalamus, pituitary and medial OFC; (ii) lower BGU after eating in cuneus, parietal lobule, superior and middle temporal gyrus, occipital pole, precuneus, PCC, and angular gyrus than the two other groups; (iii) lower BGU in insula, dlPFC, lateral OFC, frontal operculum cortex and anterior medial frontal cortex than participants with obesity (but not normal-weight participants); and (iv) a lower BGU in lingual gyrus than group with normal weight (but not obesity). Acute administration of somatostatin (and insulin) to suppress the heightened post-prandial satiety gut hormones (including plasma PYY and GLP-1) in the post-RYGB surgery group had no effect on post-prandial BGU in any of the fROIs (determined from regions showing an interaction between group and nutritional state from the results above), except for an attenuated post-prandial increase in BGU in the medial OFC (but no correction was done for multiple comparisons)

Two longitudinal studies with mixed RYGB/VSG surgery groups examined the effects of hyeprinsulinaemic euglycaemic clamps (vs. fasting). RYGB/VSG surgery had no effect on insulin-stimulated BGU at six months (vs. pre-surgery) in whole brain analysis (n=17) (Tuulari et al. 2013). However, in aROIs analysis, insulin-stimulated BGU in midbrain, cerebellum, and iambic, frontal, parietal, temporal and occipital lobes pre-RYGB/VSG but not post-RYGB/VSG surgery (though no direct comparison between visits was reported). By contrast, in the other study, insulin-stimulated whole brain BGU decreased six months after RYGB/VSG surgery, but results for individual brain regions were not reported (n=16) (Rebelos et al. 2019). However, whole brain insulin-stimulated BGU remained higher both pre- and post-RYGB/VSG surgery compared with participants without obesity.

A longitudinal study of LCD on BGU in the fasted state, examined the influence of low or high ‘food addiction’ using the Yale Food Addiction Scale (YFAS) using aROIs analysis (n=11-12) (Guzzardi et al. 2018). In the high-YFAS group after 3 months of LCD, BGU in response to visual, taste and odour food stimuli decreased in caudate, thalamus, hippocampus, hypothalamus, midbrain, posterior central gyrus, temporal lobe, occipital lobe, with a trend for decrease in putamen, but no change in PFC, dlPFC or OFC. In the low-YFAS group there was no change in BGU in any aROIs (though no direct statistical comparison of effect of LCD intervention was made between groups).

In a very small, longitudinal study of three weeks in men with obesity, total fasting, BGU decreased in all aROIs (white matter, basal and cortical grey matter, caudate/putamen, thalamus/hypothalamus, frontal lobe, temporal lobe, occipital lobe) (n=4) (Redies et al. 1989).

***3.5.7. Brain fatty acid uptake***

*^18^F-FTHA:* One longitudinal study found no change in total FFA uptake six months post-RYGB/VSG surgery when fasted, in whole brain analysis nor ROIs analysis (frontal, parietal, temporal, occipital or limbic lobes, midbrain, or cerebellum) (n=21) (Rebelos et al. 2020). However, there was higher FFA uptake in all ROIs in obesity pre-surgery compared with controls without obesity.

By contrast, one longitudinal study found a significant reduction in total FFA uptake after six weeks of VLCD followed by 1 week of isocaloric diet, in metabolic syndrome with overweight/obesity (BMI= 33.6 ± 4 kg/m^2^) when fasted in whole brain analysis in the prefrontal cortex and parietal, temporal and occipital lobes, in grey matter, and in average of all aROIs (white matter, striatum, amygdala, hippocampus, hypothalamus, anterior cingulate cortex, prefrontal cortex, parietal and temporal lobes) (n=16) (Karmi et al. 2010). There was a higher total FFA uptake at baseline pre-VLCD compared with controls without metabolic syndrome/overweight (BMI 26.8 ± 2.5 kg/m^2^) in grey matter and average of all aROIs (Karmi et al. 2010).

*11C-palmitate:* The same study also used 11C-palmitate to examine changes in the non-oxidised fraction of FFA uptake (Karmi et al. 2010). However, no change in fractional FFA uptake rate was seen after VLCD in grey matter, or in average of all or any individual aROIs (white matter, striatum, amygdala, hippocampus, hypothalamus, anterior cingulate cortex, prefrontal cortex, parietal and temporal lobes). Conversely, as with ^18^F-FTHA uptake, there was a higher fractional FFA uptake at baseline pre-VLCD compared with participants without obesity controls without metabolic syndrome in grey matter, and in average of all and any individual aROIs (Karmi et al. 2010).

**3.6. Correlations**

***3.6.1. Clinical outcomes***

The results of the correlations with clinical outcomes from individual studies are summarised in Supplementary Table S6

*Correlations of baseline PET/SPECT measures with clinical outcomes*

*Serotonin system (pre-RYGB):* Higher baseline 5-HT_2A_R ^18^F-altanserin BP in the neocortex was associated with weight loss at ~8 months after RYGB surgery in aROIs analysis, but no correlation was seen for baseline 5-HTT ^11^C-DASB BP (n=13-21) (Haahr et al. 2015).

*Noradrenaline system (pre-LCD):* Higher baseline NAT ^11^C-MRB BP in the putamen, hippocampus, midbrain, insula and dlPFC in whole brain analysis (uncorrected for multiple comparisons) was associated with less weight loss at 6 months after LCD (n=10) (Vettermann et al. 2018).

*BGU (pre-RYGB/VSG):* Higher baseline insulin‐stimulated BGU in whole brain analysis (coordinates not given) was associated with a greater decrease in fasting plasma glucose at two years (n=17) and three years (n=13) after RYGB/VSG surgery, which persisted after correcting for baseline BMI for the two year data (Rebelos et al. 2019). By contrast, baseline BGU was unrelated to changes in BMI or HbA1c at two years after RYGB/VSG surgery (Rebelos et al. 2019).

*FFA (pre-RYGB/VSG):* Moreover, higher baseline total brain FFA uptake was associated with a lower decrease in fasting plasma glucose at two years after RYGB/LVSG surgery, and this remained significant after adjusting for baseline plasma glucose (n=21) (Rebelos et al. 2020).

*Correlations of change in PET/SPECT outcomes with change in clinical outcomes*

*Dopamine system (post-RYGB):* No correlation was seen between the increase in DRD2/3 ^123^I-IBZM BP in a striatum aROIs and the decrease in BMI, fat percent or fasting plasma glucose at 3.1 years post-RYGB surgery (n=11) (van der Zwaal et al. 2016).

*Dopamine system (post-VLCD):* A greater reduction in DRD2/3R ^18^F-fallypride BP in the substantia nigra, with a similar trend for caudate and putamen (but not hypothalamus nor ventral striatum), was associated with a greater reduction in fasting plasma glucose at 8-10 after VLCD (n=15) (Dunn et al. 2017).

*Serotonin system (post-RYGB):* A greater increase in 5-HT_2A_R ^18^F-altanserin BP in whole neocortex and in 5-HTT ^11^C-DASB averaged across all aROIs (caudate, putamen, thalamus) was associated with smaller weight loss at ~8 months after RYGB surgery (n=13-21), although there was no overall change in ^18^F-altanserin nor ^11^C-DASB BP (Haahr et al. 2015). Not CORRECTED

*Noradrenaline system (post-LCD):* A greater increase in NAT ^11^C-MRB BP in the insula and hippocampus (but not ventral striatum, caudate, putamen, thalamus, amygdala, hypothalamus, midbrain, locus coeruleus, ACC, mid prefrontal cortex, dlPFC, OFC) was associated with less weight loss at 6 months post-LCD (n=10) (though this analysis was not fully corrected for multiple corrections), although there was no overall change in ^11^C-MRB BP after LCD in any region (Vettermann et al. 2018).

*Brain glucose uptake (post-LCD):* The change in BGU upon HE food presentation in any aROIs (caudate putamen, thalamus, hippocampus, hypothalamus, midbrain, prefrontal cortex, dlPFC, OFC, post-central gyrus, temporal and occipital lobes) did not correlate with weight loss, body fat percentage, subcutaneous and visceral adipose tissue volumes at three months after LCD (1600kcal per day) in either groups with high or low YFAS questionnaire score (n=11-12) (Guzzardi et al. 2018).

***3.6.2. Behavioural outcomes***

Behavioural measures and the association with PET/SPECT findings are summarised in Supplementary Tables S7 and S8

*Correlations of changes in PET/SPECT measures with changes in behavioural outcomes*

*Dopamine system*

*Post-RYGB:* A smaller increase in DRD2/3 BP using ^123^I-IBZM in the striatum aROIs tended to be associated with a greater decrease in general food craving trait (but not state) questionnaire at 3.1 years post-RYGB surgery, despite on average the DRD2/3 BP increasing and the food craving decreasing after RYGB surgery (van der Zwaal et al. 2016).

*Serotonin system*

*Post-RYGB****:*** Neither the change in 5-HT_2A_R BP using 18F-altanserin tracer in neocortex, nor change in SERT BP using ^11^C-DASB tracer in average caudate, putamen and thalamus aROIs, correlated with the increase in post-prandial fullness using visual analogue scales at 8 months post-RYGB surgery (Haahr et al. 2015).

*Brain glucose uptake*

*Post-LCD:* In one longitudinal study, many correlations (uncorrected for multiple comparisons) were made between BGU using ^15^O-H_2_O PET and YFAS score, hunger ratings, pleasantness of visual, olfactory and gustatory food cues, and dietary recall of oligosaccharide, lipid and cholesterol intake, at either baseline or post-LCD in participants with overweight and either low or high baseline YFAS scores (Guzzardi et al. 2018). However, no predictive correlations were done to examine whether baseline BGU predicted changes in eating behaviour, nor whether changes in BGU correlated with changes in eating behaviour, after the LCD intervention.

*Post-RYGB surgery:* A cross-sectional study examined correlations between changes in glucose uptake using ^18^F-FDG after food intake (fed-fasted) with *ad libitum* energy intake when fasted separately in individual groups: post-RYGB, participants with obesity or normal weight (Hunt et al. 2016). In the normal weight group, the post-prandial change in BGU in the right dlPFC positively correlated with energy intake when fasted, with greater decrease in BGU associated with lower *ad libitum* consumption; while post-prandial changes in BGU in subcallosal gyrus and hypothalamus negatively correlated with energy intake with greater increase in BGU associated with lower *ad libitum* consumption. These positive and negative correlations were also seen in the group post-RYGB surgery, but not the unoperated group with obesity. In both the groups post-RYGB surgery and with unoperated obesity, the post-prandial change in BGU in the right angular gyrus, left parietal lobule, occipital pole and posterior right STG and MTG were also positively correlated with energy intake when fasted. In the group post-RYGB surgery, but not the groups with normal weight or obesity, the post-prandial change in BGU in right medial OFC, posterior cingulate cortex, precuneus and cuneus were positively correlated with energy intake when fasted. However, direct comparison of correlations between the groups was not performed.

Correlations of post-prandial changes in visual analogue scale ratings of fullness and sickness with post-prandial changes in BGU were only reported across all three groups combined, and so the individual effects of RYGB surgery were not assessed (Hunt et al. 2016).

*Regional cerebral blood flow*

*Post-LCD:* No cross-sectional correlations were seen between the effect of food intake or tasting of HE food on CBF using ^15^O-H_2_O PET in any fROIs (amygdala, posterior hippocampus, mid insula, PCC) and fed state hunger or fullness ratings in post-LCD, participants with obesity or normal weight groups (Delparigi et al. 2004).

In a combined analysis of participants post-LCD and participants with obesity without intervention, the effects of food intake (fed-fasted) on ^15^O-H_2_O PET in cerebellum and dorsal PFC fROIs were positively correlated, and in OFC negatively correlated (but not putamen, and without correction for multiple comparisons) with TFEQ-dietary restraint, but not disinhibition or hunger-related eating (DelParigi et al. 2007).

***3.6.3. Mood assessment***

Mood assessment and the association with PET/SPECT findings are summarised in Supplementary Table S7 and S8.

*Correlations of PET/SPECT measures* *with mood*

*Opioid system*: Burghardt et al. (2015) carried out a study in lean vs. participants with obesity participants. Increment in MOR BP in temporal pole from fasted to fed state negatively correlated with larger decrease in negative affect in lean participants, while no correlation was observed in participants with obesity pre- or post-VLCD.

***3.6.4. Mechanistic outcomes***

Blood mechanistic measures and the association with PET/SPECT findings are summarised in Supplementary Table S9 and S10

*Correlations of changes in PET/SPECT measures with changes in mechanistic outcomes*

*Dopamine system:*

*Post-RYGB:* The increase in DRD2/3 BP using ^123^I-IBZM in the striatum aROIs at 3.1 years post-RYGB did not correlate with the decrease in fasting plasma/serum leptin, total ghrelin, insulin, or QUICKI measure of insulin resistance (van der Zwaal et al. 2016).

*Post-VLCD:* A greater decrease in DRD2/3 BP using ^18^F-fallypride in the ventral striatum, putamen, caudate, hypothalamus and substantia nigra in aROI analysis was associated with a greater decrease in fasting plasma leptin at 8-10 days post-VLCD, but no correlation was seen with percentage change in fasting insulin, insulin disposition index or plasma acyl ghrelin (Dunn et al. 2017).

*Serotonin system:*

*Post-RYGB****:*** The change in 5-HT_2A_R BP in neocortex using ^18^F-altanserin, or SERT BP averaged across caudate, putamen and thalamus aROIs using ^11^C-DASB, did not correlate with the increase in post-prandial (400 kcal) area under curve (AUC) total GLP-1 at 8 months post-RYGB (Haahr et al. 2015).

*Cross-sectional correlations of PET/SPECT measures* *with mechanistic outcomes*

Some cross-sectional studies examined correlations between the effects of food intake on plasma metabolites, insulin and gut hormones, and effects of food intake on rCBF using ^15^O-H_2_O PET across groups (post-VLCD, participants with obesity or normal weight) (DelParigi et al. 2007), or glucose uptake using ^18^F-FDG across groups (post-RYGB, participants with obesity or normal weight) (Hunt et al. 2016), but this was not helpful in examining the role for these mechanistic factors on differences in PET measures between intervention groups.

**SUPPLEMENTARY REFERENCES**

Almby K.E., Lundqvist M.H., Abrahamsson N., Kvernby S., Fahlstrom M., Pereira M.J., Gingnell M., Karlsson F.A., Fanni G., Sundbom M., Wiklund U., Haller S., Lubberink M., Wikstrom J., Eriksson J.W., 2021. Effects of gastric bypass surgery on the brain; simultaneous assessment of glucose uptake, blood flow, neural activity and cognitive function during normo- and hypoglycemia. Diabetes. 70, 1265-1277.

Burghardt P.R., Rothberg A.E., Dykhuis K.E., Burant C.F., Zubieta J.K., 2015. Endogenous Opioid Mechanisms Are Implicated in Obesity and Weight Loss in Humans. J Clin Endocrinol Metab. 100, 3193-3201.

de Weijer B.A., van de Giessen E., Janssen I., Berends F.J., van de Laar A., Ackermans M.T., Fliers E., la Fleur S.E., Booij J., Serlie M.J., 2014. Striatal dopamine receptor binding in morbidly obese women before and after gastric bypass surgery and its relationship with insulin sensitivity. Diabetologia. 57, 1078-1080.

Delparigi A., Chen K., Salbe A.D., Hill J.O., Wing R.R., Reiman E.M., Tataranni P.A., 2004. Persistence of abnormal neural responses to a meal in postobese individuals. Int J Obes Relat Metab Disord. 28, 370-377.

DelParigi A., Chen K., Salbe A.D., Hill J.O., Wing R.R., Reiman E.M., Tataranni P.A., 2007. Successful dieters have increased neural activity in cortical areas involved in the control of behavior. Int J Obes (Lond). 31, 440-448.

Dunn J.P., Abumrad N.N., Kessler R.M., Patterson B.W., Li R., Marks-Shulman P., Tamboli R.A., 2017. Caloric Restriction-Induced Decreases in Dopamine Receptor Availability are Associated with Leptin Concentration. Obesity. 25, 1910-1915.

Dunn J.P., Cowan R.L., Volkow N.D., Feurer I.D., Li R., Williams D.B., Kessler R.M., Abumrad N.N., 2010. Decreased dopamine type 2 receptor availability after bariatric surgery: preliminary findings. Brain Res. 1350, 123-130.

Guzzardi M.A., Garelli S., Agostini A., Filidei E., Fanelli F., Giorgetti A., Mezzullo M., Fucci S., Mazza R., Vicennati V., Iozzo P., Pagotto U., 2018. Food addiction distinguishes an overweight phenotype that can be reversed by low calorie diet. Eur Eat Disord Rev. 26, 657-670.

Haahr M.E., Hansen D.L., Fisher P.M., Svarer C., Stenbaek D.S., Madsen K., Madsen J., Holst J.J., Baare W.F., Hojgaard L., Almdal T., Knudsen G.M., 2015. Central 5-HT neurotransmission modulates weight loss following gastric bypass surgery in obese individuals. J Neurosci. 35, 5884-5889.

Hunt K.F., Dunn J.T., le Roux C.W., Reed L.J., Marsden P.K., Patel A.G., Amiel S.A., 2016. Differences in regional brain responses to food ingestion after Roux-en-Y gastric bypass and the role of gut peptides: a neuroimaging study. Diabetes Care. 39: 1787-1795.

Karlsson H.K., Tuulari J.J., Tuominen L., Hirvonen J., Honka H., Parkkola R., Helin S., Salminen P., Nuutila P., Nummenmaa L., 2016. Weight loss after bariatric surgery normalizes brain opioid receptors in morbid obesity. Mol Psychiatry. 21, 1057-1062.

Karmi A., Iozzo P., Viljanen A., Hirvonen J., Fielding B.A., Virtanen K., Oikonen V., Kemppainen J., Viljanen T., Guiducci L., Haaparanta-Solin M., Nagren K., Solin O., Nuutila P., 2010. Increased brain fatty acid uptake in metabolic syndrome. Diabetes. 59, 2171-2177.

Le D.S., Pannacciulli N., Chen K., Salbe A.D., Del P.A., Hill J.O., Wing R.R., Reiman E.M., Krakoff J., 2007. Less activation in the left dorsolateral prefrontal cortex in the reanalysis of the response to a meal in obese than in lean women and its association with successful weight loss. Am J Clin Nutr. 86, 573-579.

Marques E.L., Halpern A., Corrêa Mancini M., de Melo M.E., Horie N.C., Buchpiguel C.A., Martins Novaes Coutinho A., Ono C.R., Prando S., Santo M.A., Cunha-Neto E., Fuentes D., Cercato C., 2014. Changes in neuropsychological tests and brain metabolism after bariatric surgery. J Clin Endocrinol Metab. 99, E2347-2352.

Rebelos E., Hirvonen J., Bucci M., Pekkarinen L., Nyman M., Hannukainen J.C., Iozzo P., Salminen P., Nummenmaa L., Ferrannini E., Nuutila P., 2020. Brain free fatty acid uptake is elevated in morbid obesity, and is irreversible 6 months after bariatric surgery: A positron emission tomography study. Diabetes Obes Metab. 22, 1074-1082.

Rebelos E., Immonen H., Bucci M., Hannukainen J.C., Nummenmaa L., Honka M.J., Soinio M., Salminen P., Ferrannini E., Iozzo P., Nuutila P., 2019. Brain glucose uptake is associated with endogenous glucose production in obese patients before and after bariatric surgery and predicts metabolic outcome at follow-up. Diabetes Obes Metab. 21, 218-226.

Redies C., Hoffer L.J., Beil C., Marliss E.B., Evans A.C., Lariviere F., Marrett S., Meyer E., Diksic M., Gjedde A., et al., 1989. Generalized decrease in brain glucose metabolism during fasting in humans studied by PET. Am J Physiol. 256, E805-810.

Steele K.E., Prokopowicz G.P., Schweitzer M.A., Magunsuon T.H., Lidor A.O., Kuwabawa H., Kumar A., Brasic J., Wong D.F., 2010. Alterations of central dopamine receptors before and after gastric bypass surgery. Obes Surg. 20, 369-374.

Tuulari J.J., Karlsson H.K., Hirvonen J., Hannukainen J.C., Bucci M., Helmio M., Ovaska J., Soinio M., Salminen P., Savisto N., Nummenmaa L., Nuutila P., 2013. Weight loss after bariatric surgery reverses insulin-induced increases in brain glucose metabolism of the morbidly obese. Diabetes. 62, 2747-2751.

van der Zwaal E.M., de Weijer B.A., van de Giessen E.M., Janssen I., Berends F.J., van de Laar A., Ackermans M.T., Fliers E., la Fleur S.E., Booij J., Serlie M.J., 2016. Striatal dopamine D2/3 receptor availability increases after long-term bariatric surgery-induced weight loss. Eur Neuropsychopharmacol. 26, 1190-1200.

Versteeg R.I., Schrantee A., Adriaanse S.M., Unmehopa U.A., Booij J., Reneman L., Fliers E., la Fleur S.E., Serlie M.J., 2017. Timing of caloric intake during weight loss differentially affects striatal dopamine transporter and thalamic serotonin transporter binding. FASEB Journal. 31, 4545-4554.

Vettermann F.J., Rullmann M., Becker G.A., Luthardt J., Zientek F., Patt M., Meyer P.M., McLeod A., Brendel M., Blüher M., Stumvoll M., Hilbert A., Ding Y.S., Sabri O., Hesse S., 2018. Noradrenaline transporter availability on [(11)C]MRB PET predicts weight loss success in highly obese adults. Eur J Nucl Med Mol Imaging. 45, 1618-1625.
